# Crystallizing covalent organic frameworks from metal organic framework through chemical induced-phase engineering

**DOI:** 10.1038/s41598-023-46573-3

**Published:** 2023-11-09

**Authors:** Abdul Khayum Mohammed, Safa Gaber, Jésus Raya, Tina Skorjanc, Nada Elmerhi, Sasi Stephen, Pilar Pena Sánchez, Felipe Gándara, Steven J. Hinder, Mark A. Baker, Kyriaki Polychronopoulou, Dinesh Shetty

**Affiliations:** 1 Department of Chemistry, Khalifa University, PO Box: 127788, Abu Dhabi, United Arab Emirates; 2 Membrane Biophysics and NMR, Institute of Chemistry, University of Strasbourg – CNRS, Rue BlaisePascal 1, Strasbourg, France; 3 Materials Research Laboratory, University of Nova Gorica, Vipavska 11C, 5270 Ajdovscina, Slovenia; 4 Instituto de Ciencia de Materiales de Madrid-CSIC, C/Sor Juana Inés de La Cruz 3, 28049 Madrid, Spain; 5 The Surface Analysis Laboratory, Engineering and Physical Sciences, University of Surrey, Guildford, GU2 4DL UK; 6 Center for Catalysis and Separations, Khalifa University, PO Box: 127788, Abu Dhabi, United Arab Emirates

**Keywords:** Chemistry, Materials science

## Abstract

The ordered porous frameworks like MOFs and COFs are generally constructed using the monomers through distinctive metal-coordinated and covalent linkages. Meanwhile, the inter-structural transition between each class of these porous materials is an under-explored research area. However, such altered frameworks are expected to have exciting features compared to their pristine versions. Herein, we have demonstrated a chemical-induction phase-engineering strategy to transform a two-dimensional conjugated Cu-based SA-MOF (Cu-Tp) into 2D-COFs (Cu-TpCOFs). The structural phase transition offered in-situ pore size engineering from 1.1 nm to 1.5–2.0 nm. Moreover, the Cu-TpCOFs showed uniform and low percentage-doped (~ 1–1.5%) metal distribution and improved crystallinity, porosity, and stability compared to the parent Cu-Tp MOF. The construction of a framework from another framework with new linkages opens interesting opportunities for phase-engineering.

## Introduction

The structural phase transition of solids involves several physicochemical changes including lattice transformations of crystalline phases^1–3^. The external triggers such as light, electricity, temperature, pressure, and chemical induction result in slight modification or full transformations of the structure of crystalline solids^4–14^. Importantly, structural phase engineering is recognized and explored in extended molecular materials such as metal–organic frameworks (MOFs)^15–17^. MOFs are crystalline porous solids, composed of organic linkers and metal knots linked through coordination bonds^18,19^. The fine regulation over the structure of MOFs through phase engineering opened the window of functionally diverse framework architectures^20^. Notably, phase engineering of MOFs alters or modifies their building blocks, lattices, porosity, etc. Moreover, the investigations on phase-engineering of MOFs provide the opportunity for understanding the structure–property correlation of materials. However, the reported phase engineering of MOFs mostly dealt with the physical transformation of the crystalline phases. The primary coordination bonds between metal knots and organic linkers are preserved in every case of such transformations. The replacement of coordinated metals in the framework into covalently bonded organic linkers has never been reported before. This type of phase engineering results in a complete transformation of MOFs into another class of porous materials, called covalent organic frameworks (COFs)^21–26^, the crystalline and porous organic solids linked by symmetric organic linkers. The inter-class transformation of crystalline porous materials is a rarely explored research area and has the potential to provide more opportunities to understand the fine physicochemical tuning of the transformed material. The phase-engineering of a MOF into a COF may provide a strong chemically-linked framework with uniform doping of metals. In general, COFs are lightweight and chemically more stable materials than MOFs. However, such conversions are not well explored due to the lack of functional availability for chemical conversion. Crystallizing COFs from MOFs confronts a major challenge of chemical transformation. The COFs are crystallized through dynamic covalent chemistry (DCC) by utilizing specific functional groups of monomers to form chemical bonds^27^. The absence of such functional groups in MOFs restricts their conversion into COFs.

Keeping all these aspects, herein, we introduce a novel chemical induction strategy to crystallize metal-doped two-dimensional (2D)-COFs through the phase-engineering of 2D-MOFs. The phase-engineering of MOFs allowed a uniform, and modest distribution of copper in the transformed COFs due to the metal interaction with polar bonds (C=O, C=N, C=C–N) within the 2D COFs. Firstly, we have developed a novel class of conjugated MOFs, called salicylaldehydate MOFs (SA-MOFs) from *1, 3, 5*-triformylphloroglucinol (Tp)^28^. In this class of MOFs, the salicylaldehyde functional pockets in Tp are coordinated with metal ions to form the 2D framework. Interestingly, the introduction of protonated C_2_/C_3_ symmetric amine linkers (*4, 4*-Azodianinile (Azo) or *4, 4', 4'*'-(*1, 3, 5*-Triazine-*2, 4, 6*-triyl)trianiline (Tta)) into the copper-linked SA-MOF (Cu-Tp) results in its physicochemical transformation into 2D-COFs (Cu-TpAzo and Cu-TpTta). During the reaction, the functional flipping (for connecting the linker) of aldehyde in Tp from nucleophilic oxygen to electrophilic carbon is the critical step in the conversion of coordination to a covalent bond. Notably, the Cu-TpAzo and Cu-TpTta COFs showed improved crystallinity, porosity, and chemical stability compared to Cu-Tp.

There are two ways to incorporate metals in COFs: (1) ex-situ or in-situ metal doping and (2) the integration of metal-anchored symmetric linkers. The former method leads to the non-uniform distribution of metals whereas the latter method offers a uniform distribution of metals but is limited to specific metal-coordinated linkers. Interestingly, the phase-engineering strategy provides the opportunity for uniform distribution of metals irrespective of the amine linkers (Azo or Tta) used for the transformation. The copper atoms are symmetrically organized in the parent Cu-Tp which results in an overall uniform and imperceptible distribution of copper in COFs.

## Result and discussion

**Figure 1:**
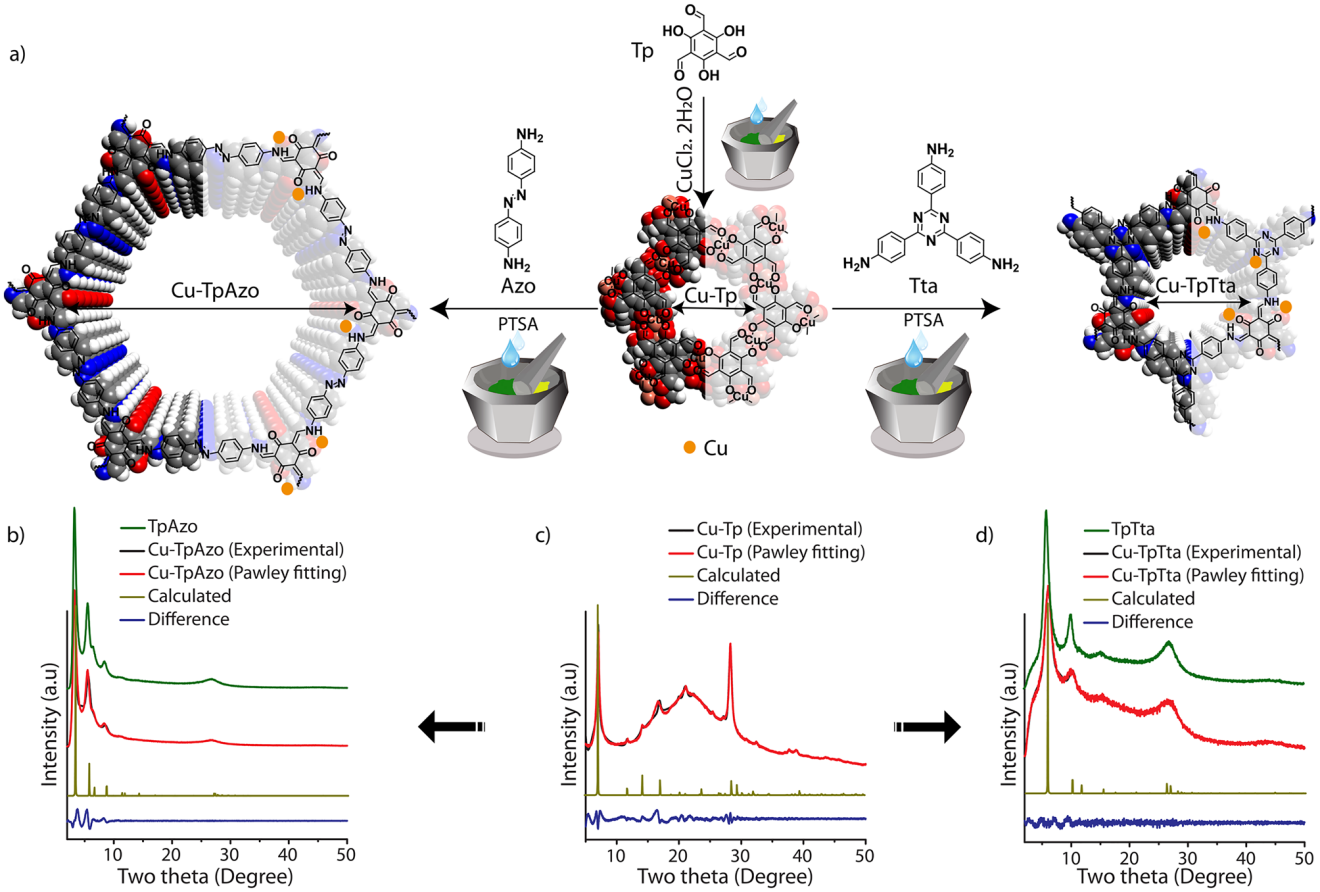
(**a**) The schematic representation and theoretical models of the chemical-induced phase-engineering of Cu-Tp into Cu-TpAzo and Cu-TpTta. The PXRD profiles of (**b**) Cu-TpAzo; (**c**) Cu-Tp and (**d**) Cu-TpTta.

The Cu-Tp MOF was synthesized by the previously reported procedure^28^. The phase engineering of Cu-Tp was carried out through the direct addition of a protonated amine to the MOF and subsequent mechanomixing and thermal treatment (Fig. 1a; Synthetic details, SI). Herein, the amine linkers (Azo and Tta) were protonated using p-toluenesulphonic acid (PTSA). The color of Cu-Tp was changed from dark green into either dark brown or light green upon mixing with Azo-PTSA and Tta-PTSA, respectively. The thermal treatment of the mixture (90 °C, 24 h) and subsequent washing with *N, N*-dimethylacetamide (DMA), water, and acetone yielded a reddish color Cu-TpAzo and yellow color Cu-TpTta (Fig. S1). Notably, the chemical induction causes physicochemical changes during the MOF to COF transformation. The color transition and abrupt change in the physical texture of the material are visible observations of phase engineering.

The powder-X-ray diffraction (PXRD) profiles revealed the ordered structural transformation of 2D-MOF into 2D-COFs (Fig. 1b–d). While Cu-Tp showed major peaks at two theta ~ 7.1°, ~ 14.0°, ~ 16.6°, ~ 21.1°, and ~ 28.2°, Cu-TpAzo and Cu-TpTta showed clear phase modification after the chemical induction by displaying sharp crystalline peaks at two thetas 3.2°, 5.5°, 6.5°, 8.4°, and 26.7°, and 6.0°, 10.0°, and 26.7°, respectively. The PXRD profiles of Cu-TpAzo and Cu-TpTta match with the pristine TpAzo and TpTta. These observations signify the hexagonal AA stacking structure of phase-engineered COFs^29,30^. The disappearance of Cu-Tp characteristic peaks indicates its full transformation into a new crystalline phase.

**Figure 2:**
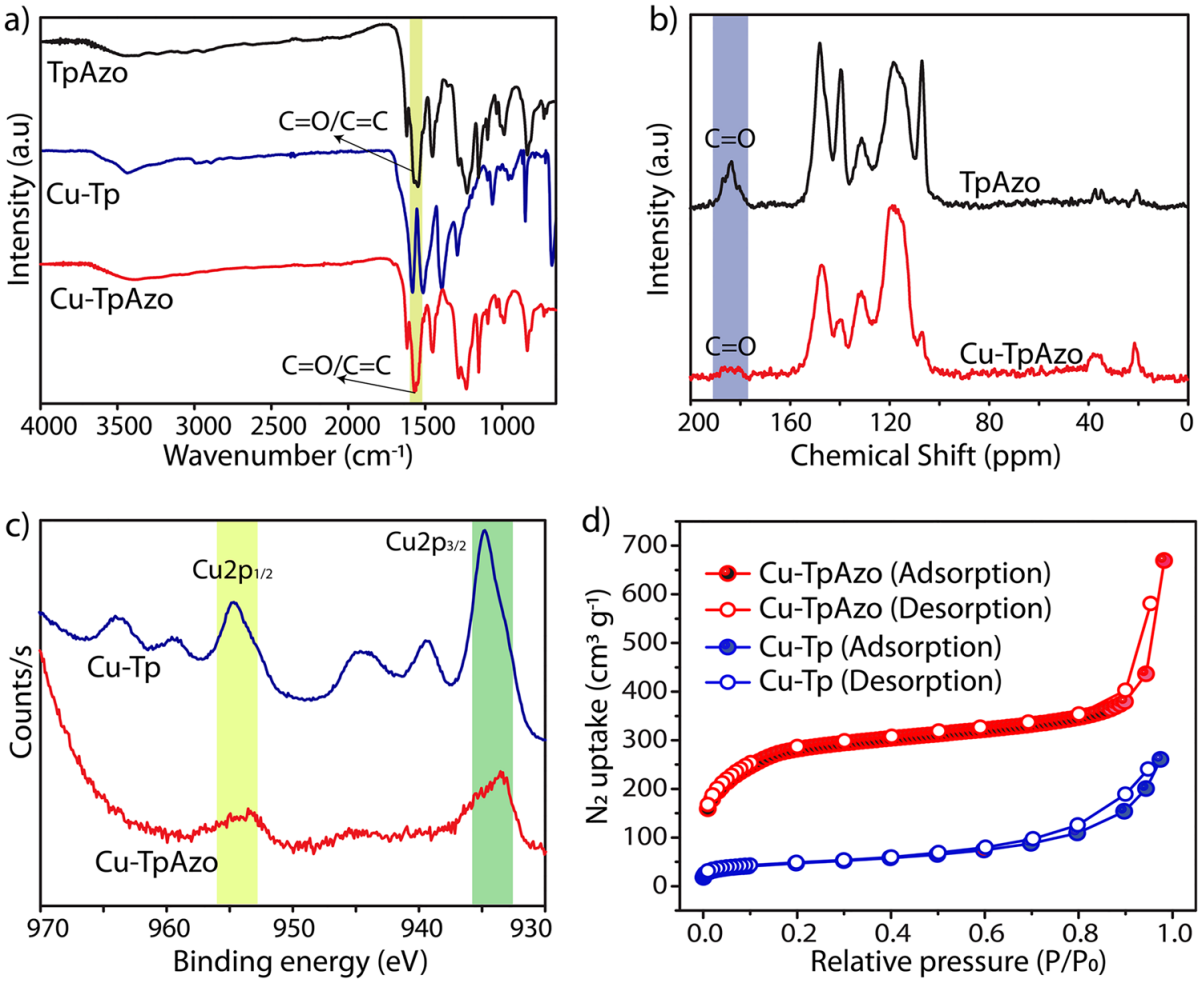
(**a**) FT-IR profiles of Cu-TpAzo, TpAzo, and Cu-Tp. (**b**) ^13^C CP-MAS solid-state NMR profiles of Cu-TpAzo and TpAzo. (**c**) XPS profiles of Cu-TpAzo and Cu-Tp. (**d**) N_2_ gas adsorption isotherms profiles of Cu-TpAzo and Cu-Tp.

The chemical bonding transformations of Cu-Tp to Cu-TpAzo/Tta were recorded using FT-IR spectroscopy (Fig. 2a; Figs. S2–S3). The transformation of functional groups from coordination to covalent bonds can significantly alter the corresponding stretching frequencies: the coordinated C=O in Cu-Tp stretched at the frequency of 1550 cm^−1^, whereas the FT-IR profiles of Cu-TpAzo and Cu-TpTta match with the pristine TpAzo and TpTta which signifies the overall similarity in the chemical environment. The Cu-TpAzo showed stretching peaks at 1570 and 1550 cm^−1^ corresponding to the C=O and C=C bonds, respectively, originating from the *β*-ketoenol tautomerism^31^. Similarly, the stretching frequencies of C=O and C=C in Cu-TpTta were observed at 1596 and 1570 cm^−1^, respectively. Notably, Cu-TpAzo and Cu-TpTta showed a slight peak shift towards higher frequency for C=O compared to their pristine COFs. This could be due to the interactions with the residual copper ions present in the framework. The newly formed C–N stretching peaks at 1224 cm^−1^ (Cu-TpAzo) and 1254 cm^−1^ (Cu-TpTta) confirm the chemical conversion of Cu-Tp into COFs.

Furthermore, ^13^C CP MAS solid-state NMR spectra of Cu-TpAzo and Cu-TpTta unfolded the atomic-level details of the phase-engineered frameworks (Fig. 2b; Fig. S4). The chemical shift peaks of Cu-TpAzo and Cu-TpTta matched with those in TpAzo and TpTta which confirms the transformation of coordination bonds into covalent bonds. Importantly, both Cu-TpAzo and Cu-TpTta showed the nitrogen-bonded *sp*^2^ carbon at 147 ppm which is in good agreement with TpAzo and TpTta. It also signifies the chemical organization of the phase-engineered COFs in *β*-ketoenol tautomerism. Moreover, the aromatic *sp*^*2*^ carbons are present from 117 to 140 ppm and 112 to 140 ppm for Cu-TpAzo and Cu-TpTta, respectively. Interestingly, the peak intensity variations were noted in the polar bonds (‒*C=O, ‒*C=N‒, ‒HN‒*C=*C‒) of both phase-engineered COFs compared to the pristine COFs. Notably, unlike the TpAzo and TpTta, the intensity of C=O (at 183 ppm) was diminished in both Cu-TpAzo and Cu-TpTta. Also, peak intensities of both *sp*^*2*^ carbons of exocyclic C=C in Tp were retarted with respect to peak intensities of aromatic *sp*^2^ carbons. Particularly, the reduction of peak intensity was also noted in the C=N in the triazine core of Cu-TpTta. The plausible reason for the reduction of peak intensities of polar bonds could be due to the interaction with residual copper ions present in the material. These interactions could lead to the pulling of electrons from the conjugated π bonds (O=C–C=C-) towards oxygen. In turn, the copper interaction could lead to the partial redistribution of electrons from the conjugated π bonds towards oxygen, causing a partial disruption of polarized double bonds in the system (Fig. S6b). The inductively coupled plasma mass spectrometry (ICP-MS) suggested the lower concentrations of Cu in Cu-TpAzo (~ 1.8%) and Cu-TpTta (1.3%). The overall peak intensity reduction in NMR with a lower amount of copper signifies their uniform distribution throughout the framework as a result of framework-to-framework transformation.

The X-ray photoelectron spectroscopy (XPS) validated the transformation in the chemical state of elements from Cu-Tp to the corresponding COFs (Fig. 2c and Fig. S7). The C1s profiles of Tp and Cu-Tp showed higher binding energy peaks at 287.1 eV and 287.4 eV, respectively. These higher binding energy peaks could originate from the *C=O. In comparison to Tp and Cu-Tp, the C1s profile of Cu-TpAzo and Cu-TpTta showed significant diminishing and lower binding energy shifts of these peaks to 286.2 eV and 286.8 eV, respectively. This could be due to the transition of the C=O into C–N (from β-ketoenamine framework). Moreover, the chemical state of carbon in the phase-engineered (Cu-TpAzo and Cu-TpTta) and direct-synthesized COFs (TpAzo and TpTta) showed a similar binding energy profile. Again, the binding energy shifts of O1s and N1s of Cu-TpAzo and Cu-TpTta are also closer to the direct-synthesized COFs. The O1s of Cu-TpAzo and Cu-TpTta showed slight variations in the intensity of the peaks which could be due to the possible weak interaction with copper. Notably, the presence of copper was also confirmed through XPS analysis. The binding energy mapping of copper in Cu-TpAzo and Cu-TpTta signifies their chemical state in the reduced form, unlike the + 2-oxidation state in parent Cu-Tp. In detail, the Cu-Tp showed Cu2p_3/2_ (934.93 eV) and Cu2p_1/2_ (954.80 eV) with corresponding strong satellite peaks. In contrast, the Cu-TpAzo and Cu-TpTta displayed lower shift binding energy peaks of Cu2p_3/2_ (933.4 eV) and Cu2p_1/2_ (953.4 eV) with weak intensity satellite peaks, indicating the reduction of the oxidation state of copper from + 2 to + 1. The reduction of the copper oxidation state could be due to the presence of basic sites (N & O) during the chemically-induced phase engineering step. The reduction possibility of Cu^2+^ was confirmed through the XPS analysis of TpAzo + Cu. Herein, the + 2 oxidation state in the CuCl_2_·2H_2_O was reduced to a + 1 oxidation state after the reaction.

The thermogravimetric analysis (TGA) provided the thermal stability of Cu-TpAzo and Cu-TpTta up to ~ 400 °C which is higher than the Cu-Tp (~ 345 °C) (Fig. S8). Moreover, the phase-engineering of the frameworks is associated with the creation of new porous architecture from the parent porous material. Interestingly, the conversions of Cu-Tp into Cu-TpAzo and Cu-TpTta went through distinctive symmetric combinations (C_3_ + C_2_ for Cu-TpAzo and C_3_ + C_3_ for Cu-TpTta) and finally engineered the porous networks with two different pore sizes. The N_2_ adsorption isotherm at 77 K showed improved BET surface area for Cu-TpAzo (1065 m^2^ g^−1^) and moderate BET surface area for Cu-TpTta (214 m^2^ g^−1^) in comparison to Cu-Tp (BET surface area of 167 m^2^ g^−1^) (Fig. 2d and Fig. S9). The difference in symmetry and the length of amine linkers results in characteristic pore size distribution for phase-engineered COFs (Fig. S10). The pore size expansion from Cu-Tp (~ 1.1 nm) to Cu-TpAzo (~ 1.9 nm) was noted in the NLDFT pore size distribution plot. On the contrary, the pore size of Cu-TpTta showed ~ 1.5 nm. Notably, the CO_2_ adsorption at 273 K showed the uptake of 1.9 and 1.2 mmol g^−1^ for Cu-TpAzo and Cu-TpTta, respectively (Fig. S11).

**Figure 3:**
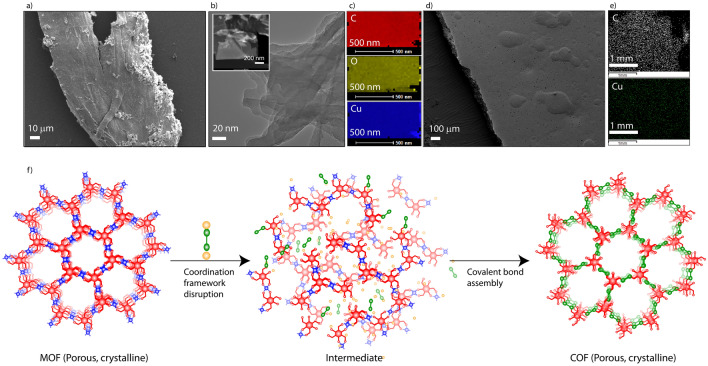
(**a**) SEM image of Cu-TpAzo. (**b**) TEM image of Cu-TpAzo (inset: elemental mapping area of Cu-TpAzo). (**c**) Elemental mapping images of Cu-TpAzo (carbon-red; oxygen-yellow; and copper-blue). (**d**) SEM image of Cu-TpAzo sheet. (**e**) SEM elemental mapping of Cu-TpAzo sheet (carbon-gray; and copper–green). (**f**) Graphical representation of the conversion of a porous and crystalline MOF into porous and crystalline COF through coordination bond disruption and covalent bond formation.

The mechano-mixing synthesized COFs often yield µm to mm size monolith particles. The scanning electron microscopy (SEM) images of Cu-TpAzo and Cu-TpTta are similar to the microstructure morphology of TpAzo and TpTta (Fig. 3a; Figs. S12–S14). Moreover, the elemental mapping showed a uniform presence of copper (~ 1%) in the materials. We could also fabricate the macroscopic and free-standing 2D sheet of Cu-TpAzo with a uniform distribution of Cu (Fig. 3d,e; Fig. S15). Again, transmission electron microscopy (TEM) images showed sheet-like nanostructure for Cu-TpAzo with the presence of Cu (Fig. 3b,c).

The phase engineering is associated with the breaking of the coordination bonds between Tp and the metal ions, and the construction of covalent bonds with new aromatic linkers (Fig. 3f). It causes the variation of the electronic arrangement in the framework, which is reflected in the visible light absorption. Solid-state UV–visible spectroscopy was used to analyze the electronic response toward the light (Fig. S16). The Cu-Tp absorbed the light energy from 750 to 490 nm with the wavelength maxima of 635 nm. Meanwhile, the Cu-TpAzo showed broad light absorption from 800 to 400 nm with a slight wavelength maximum of 580 nm. Likewise, Cu-TpTta also exhibited a broad absorption range of light with wavelength maxima of 494 nm. Moreover, the light absorption profiles of Cu-TpAzo and Cu-TpTta almost match with TpAzo and TpTta.

In Cu-Tp, the C_3_ symmetric Tp has three hydroxyls (-OH) and three aldehyde (-HC=O) groups which are coordinated to Cu^2+^ ions and form a hexagonal network^28^. Herein, the aldehyde group has two functional sites for chemical reaction, an electrophilic carbon and a nucleophilic oxygen. The Lewis acid Cu^2+^ coordinative interacted with the nucleophilic oxygen of aldehyde during the MOF formation (Fig. 2e). However, the presence of protonated amine salt demetallate the aldehyde group (proton-metal exchange) which makes it susceptible to nucleophilic attack at carbon. It should be noted that the presence of protons is required for such demetallation of aldehyde. The non-protonated amine, however, failed to transform the coordination to the covalent network. Interestingly, the chemical-induced phase-engineering of Cu-Tp transformed the chemical bond from coordination to a covalent network in the solid-state mixing state even at room temperature. The FT-IR spectra of Cu-TpAzo suggested the formation of C=O, C=C, and C–N which signifies the chemical bond transfer from MOF to COF (Fig. S17). However, the Cu-TpAzo was not completely crystallized at the room-temperature and required further thermal treatment. The absence of (100) peak (at two theta 3.2°) and the presence of (001) peak (at two theta 26.7°) in the PXRD profile of the room temperature mixture of Cu-TpAzo indicates the formation of non-stacked 2D COF layers (Fig. S18). The *ex-situ* PXRD analysis showed a gradual increment of the crystallinity of COF upon increasing the time of thermal treatment at 90 °C. The relative peak intensity of (100) has been improved from 1 to 24 h of thermal treatment signifies the role of temperature for the effective stacking of the formed 2D COF layers (Table S1; Fig. S18). Moreover, the phase-engineered COFs also showed chemical and structural stabilities in 2 M H_2_SO_4_ for 24 h. The retained crystallinity and chemical bonds in PXRD and FT-IR profiles of Cu-TpAzo and Cu-TpTta suggested the structural and chemical intactness in the acid medium (Figs. S19–S20). It is important to note that the acid treatment leads to the leaching of copper from the COF. ICP-MS analysis revealed only 0.0002% copper in Cu-TpAzo after subjecting it to 2 M H_2_SO_4_ for 24 h, suggesting the potential for producing a pure COF from the metal-doped COF.

In order to understand the significance of phase engineering, we have synthesized copper-doped TpAzo through *in-situ* addition of CuCl_2_·2H_2_O into the COF precursor mixture. The obtained COF (TpAzo + Cu) showed a lower degree of crystallinity and surface area (266 m^2^ g^−1^) compared to the Cu-TpAzo (Figs. S5 and S9). In contrast, the ^13^C NMR of TpAzo + Cu showed very similar features to pristine TpAzo (Fig. S6). Unlike Cu-TpAzo, the chemical shift and intensities of peaks of TpAzo + Cu exactly match with the TpAzo. This indicates the non-interaction nature of copper with the polar bonds of TpAzo. Furthermore, the TpAzo + Cu was not structurally stable upon the 2 M H_2_SO_4_ acid treatment for 24 h. The first peak (100) in the PXRD profile of TpAzo + Cu was diminished after the acid treatment. The possible reason could be due to the 2D layer delamination of lower crystalline TpAzo. We surmise the presence of copper in between the layers interacts with sulphate anions from acid, which decrease the π- π interaction between the 2D-COF layers. Overall, it signifies the advantages of the phase-engineering of MOFs for the construction of stable metal-doped COFs. The uniform distribution of Cu in the parent Cu-Tp may assist the judicious distribution of copper in the TpAzo without hampering its crystallinity, surface area, and structural stability.

## Conclusions

In conclusion, we report the synthesis of COFs from MOF through a chemically-induced phase-engineering method. The COFs are crystallized from the MOFs in solid-state and offer improved crystallinity, pore-engineering feasibility, and stability compared to the parent MOFs. Moreover, the phase-engineering method allowed a low-density distribution of copper which interacted with polar bonds (C=C, C=N, and C=O) in the framework. The metal-doped COFs have great potential in task-specific electro/photo-catalytic applications which are under investigation in our lab.

## Experimental section

### Synthesis of Cu-TpAzo

*4,4′*-Azodianiline (Azo, 103 mg) and *p*-toluenesulphonic acid (PTSA, 551.63 mg) are thoroughly mixed at room temperature until it becomes a viscous paste. To this homogeneous mixture, Cu-Tp (100 mg) is added and again mixed vigorously for 3–5 min. The green color of Cu-Tp was turned into reddish-brown colour. The obtained brown color paste was heated to 90 °C for 24 h under closed conditions. After the thermal treatment, the solid monoliths of Cu-TpAzo were washed with water, *N,N*-dimethylacetamide, water and acetone.

### Synthesis of Cu-TpTta

*1, 3, 5*-Triazine-*2, 4, 6*-triyl)trianiline (Tta, 86 mg) and *p*-toluenesulphonic acid (PTSA, 277 mg) are thoroughly mixed at room temperature until it becomes a viscous paste. To this homogeneous mixture Cu-Tp (74 mg) was added and again mixed vigorously for 3–5 min. The obtained yellow color paste was heated to 90 °C for 24 h under closed conditions. After the thermal treatment, the solid monoliths (yellow color) of Cu-TpTta were washed with water, *N,N*-dimethylacetamide, water, and acetone.

### Synthesis of TpAzo + Cu

*4,4′*-Azodianiline (Azo, 0.714 mmol) and *p*-toluenesulphonic acid (PTSA, 2.38 mmol) are thoroughly mixed at room temperature until it becomes a viscous paste. To this homogeneous mixture, Tp (0.476 mmol) was added and again mixed vigorously for 3–5 min. The CuCl_2_.2H_2_O (0.476 mmol) was added to the obtained mixture and thoroughly mixed by grinding. The obtained dark black-brown color paste was heated to 90 °C for 24 h under closed conditions. After the thermal treatment, the solid monoliths of TpAzo + Cu were washed with water, *N, N* -dimethylacetamide, water, and acetone.

### Synthesis of TpAzo

4,4′-Azodianiline (Azo, 103 mg) and p-toluenesulphonic acid (PTSA, 551.63 mg) are thoroughly mixed at room temperature until it becomes a viscous paste. To this homogeneous mixture, Tp (67.5 mg) is added and again mixed vigorously for 3–5 min. The obtained brown color paste was heated to 90 °C for 24 h under closed conditions. After the thermal treatment, the solid monoliths of TpAzo were washed with water, *N, N*-dimethylacetamide, water, and acetone.

### Synthesis of TpTta

1, 3, 5-Triazine-2, 4, 6-triyl)trianiline (Tta, 86 mg) and p-toluenesulphonic acid (PTSA, 277 mg) are thoroughly mixed at room temperature until it becomes a viscous paste. Tp (50.4 mg) was added to this homogeneous mixture and again mixed vigorously for 3–5 min. The obtained yellow color paste was heated to 90 °C for 24 h under closed conditions. After the thermal treatment, the solid monoliths (yellow color) of TpTta were washed with water, *N, N*-dimethylacetamide, water, and acetone.

### Supplementary Information

Supplementary Information.

## Data Availability

Supporting Information is available online and in the case of requirement one can contact the first author or corresponding author for original data.

## References

[1] Rao CNR, Rao KJ (1978). Phase Transitions in Solids: An Approach to the Study of the Chemistry and Physics of Solids.

[2] Wuttig M, Yamada N (2007). Phase-change materials for rewriteable data storage. Nat. Mater..

[3] Ge C, Liu J, Ye X, Han Q, Zhang L, Cui S, Guo Q, Liu G, Liu Y, Tao X (2018). Visualization of single-crystal-to-single-crystal phase transition of luminescent molecular polymorphs. J. Phys. Chem. C.

[4] Taniguchi T, Sato H, Hagiwara Y, Asahi T, Koshima H (2019). Photo-triggered phase transition of a crystal. Commun. Chem..

[5] Leonard AA, Diroll BT, Flanders NC, Panuganti S, Brumberg A, Kirschner MS, Cuthriell SA, Harvey SM, Watkins NE, Yu J, Wasielewski MR, Kanatzidis MG, Dichtel WR, Zhang X, Chen LX, Schaller RD (2023). Light-induced transient lattice dynamics and metastable phase transition in CH3NH3PbI3 nanocrystals. ACS Nano.

[6] Koshihara S, Ishikawa T, Okimoto Y, Onda K, Fukaya R, Hada M, Hayashi Y, Ishihara S, Luty T (2022). Challenges for developing photo-induced phase transition (PIPT) systems: from classical (incoherent) to quantum (coherent) control of PIPT dynamics. Phys. Rep..

[7] Deng J, Chang Z, Zhao T, Ding X, Sun J, Liu JZ (2016). Electric field induced reversible phase transition in Li doped phosphorene: shape memory effect and superelasticity. J. Am. Chem. Soc..

[8] Stegemann F, Stahl J, Bartsch M, Zacharias H, Johrendtb D, Janka O (2019). Temperature induced valence phase transition in intermediate-valent YbPd2Al3. Chem. Sci..

[9] Wu H, Feng Z, Pal A, Dong H, Jing C, Wang K, Zhang S, Deng W, Li S, Feng J, Chen J, Chen Y, Si J, Ge J-Y, Cao S, Chen B, Zhang J (2021). Evolution of temperature-induced isostructural phase transition in a newly grown layered FeTe2 single crystal. Chem. Mater..

[10] Wei X, Wang J (2022). Pressure-induced structural phase transition in EuNi2P2. ACS Omega.

[11] Anichtchenko DD, Errandonea D (2022). Pressure-induced structural phase transition in EuNi2P2. RSC Adv..

[12] Li X, Sun J, Shahi P, Gao M, MacDonald AH, Uwatoko Y, Xiang T, Goodenough JB, Cheng J, Zhou J (2018). Pressure-induced phase transitions and superconductivity in a black phosphorus single crystal. Proc. Natl. Acad. Sci. USA.

[13] Jin M, Sumitani T, Sato H, Seki T, Ito H (2018). Mechanical-stimulation-triggered and solvent-vapor-induced reverse single-crystal-to-single-crystal phase transitions with alterations of the luminescence color. J. Am. Chem. Soc..

[14] Liu S, Liu H, Zhou G, Li X, Wang S (2022). Water-induced crystal phase transformation of stable lead-free Cu-based perovskite nanocrystals prepared by one-pot method. Chem. Eng. J..

[15] Liu D, Liu T-F, Chen Y-P, Zou L, Feng D, Wang K, Zhang Q, Yuan S, Zhong C, Zhou H-C (2015). A reversible crystallinity-preserving phase transition in metal–organic frameworks: Discovery, mechanistic studies, and potential applications. J. Am. Chem. Soc..

[16] Lyu J, Gong X, Lee S-J, Gnanasekaran K, Zhang X, Wasson MC, Wang X, Bai P, Guo X, Gianneschi NC, Farha OK (2020). Phase transitions in metal–organic frameworks directly monitored through in situ variable temperature liquid-cell transmission electron microscopy and in situ X-ray diffraction. J. Am. Chem. Soc..

[17] Zhou X (2021). Molecular scalpel to chemically cleave metal–organic frameworks for induced phase transition. J. Am. Chem. Soc..

[18] Zhou H-C, Long JR, Yaghi OM (2012). Introduction to metal–organic frameworks. Chem. Rev..

[19] Furukawa H, Cordova KE, O’Keeffe M, Yaghi OM (2013). The chemistry and applications of metal-organic frameworks. Science.

[20] Ma C, Zheng L, Wang G, Guo J, Li L, He Q, Chen Y, Zhang H (2022). Phase engineering of metal-organic frameworks. Aggregate.

[21] Diercks CS, Yaghi OM (2017). The atom, the molecule, and the covalent organic framework. Science.

[22] Cote AP, Benin AI, Ockwig NW, O’Keeffe M, Matzger AJ, Yaghi OM (2005). Porous, crystalline, covalent organic frameworks. Science.

[23] DeBlase CR, Silberstein KE, Truong TT, Abruna HD, Dichtel WR (2013). β-Ketoenamine-linked covalent organic frameworks capable of pseudocapacitive energy storage. J. Am. Chem. Soc..

[24] Ding SY, Wang W (2013). Covalent organic frameworks (COFs): From design to applications. Chem. Soc. Rev..

[25] Khayum MA, Kandambeth S, Mitra S, Nair SB, Das A, Nagane SS, Mukherjee R, Banerjee R (2016). Chemically delaminated free-standing ultrathin covalent organic nanosheets. Angew. Chem. Int. Ed..

[26] Mohammed AK, Al Khoori AA, Addicoat MA, Varghese S, Othman I, Jaoude MA, Polychronopoulou K, Baias M, Haija MA, Shetty D (2022). Solvent-influenced fragmentations in free-standing three-dimensional covalent organic framework membranes for hydrophobicity switching. Angew. Chem. Int. Ed..

[27] Jin Y, Yu C, Denman RJ, Zhang W (2013). Recent advances in dynamic covalent chemistry. Chem. Soc. Rev..

[28] Mohammed AK (2023). Salicylaldehydate coordinated two-dimensional-conjugated metal–organic frameworks. Chem. Commun..

[29] Chandra S, Kundu T, Kandambeth S, BabaRao R, Marathe Y, Kunjir SM, Banerjee R (2014). Phosphoric acid loaded azo (−N=N−) based covalent organic framework for proton conduction. J. Am. Chem. Soc..

[30] Kandambeth S, Biswal BP, Chaudhari HD, Rout KC, Kunjattu HS, Mitra S, Karak S, Das A, Mukherjee R, Kharul UK, Banerjee R (2017). Selective molecular sieving in self-standing porous covalent-organic-framework membranes. Adv. Mater..

[31] Kandambeth S, Mallick A, Lukose B, Mane MV, Heine T, Banerjee R (2012). Construction of crystalline 2D covalent organic frameworks with remarkable chemical (acid/base) stability via a combined reversible and irreversible route. J. Am. Chem. Soc..

