# Supplementary material for: Crystallizing covalent organic frameworks from metal organic framework through chemical induced-phase engineering
Source: Sci Rep. 2023 Nov 9;13:19443. doi: 10.1038/s41598-023-46573-3 (PMC10636044; doi:10.1038/s41598-023-46573-3)
Supplement: Supplementary file 1 — Supplementary Information. [file 41598_2023_46573_MOESM1_ESM.docx]

Supporting Information

Crystallizing Covalent Organic Frameworks from Metal Organic Framework through Chemical Induced-Phase Engineering

Abdul Khayum Mohammed, Safa Gaber, Jésus Raya, Tina Skorjanc, Nada Elmerhi, Sasi Stephen, Pilar Pena Sánchez, Felipe Gándara, S. J. Hinder, M. A. Baker, Kyriaki Polychronopoulou , Dinesh Shetty*

**General information**

**General remarks**

All chemicals and solvents were commercially purchased and used without further purification. 4, 4-Azodianinile (Azo) was purchased from ‘Acros organics’.*1, 3, 5*-triformylphloroglucinol (Tp) was purchased from ‘Hygeia laboratories’. 4, 4', 4''-(1, 3, 5-Triazine-2, 4, 6-triyl)trianiline (Tta) and p-Toluenesulphonic acid (PTSA) were purchased from TCI chemicals. Copper (II) Chloride dihydrate was purchased from Sigma Aldrich.

**Instruments**

***PXRD:*** X-ray diffraction measurements were performed on Rigaku SmartLab II with Cu K_alpha (λ = 1.5405 Å) radiation source operating at 40 kV and 40 mA. Zero-background sample holders and a scattering protector on the detector side were used. The patterns were recorded with an incident slit of 0.1° over the 2theta range of 2–50° with step size = 0.03°. The scan speed was 2 degrees per minute.

***FT-IR***: Fourier transform infrared (FT-IR) spectra were obtained using a Bruker Optics ALPHA-E spectrometer with a universal Zn-Se ATR (attenuated total reflection) accessory or using a Diamond ATR (Golden Gate) in the 600-4000 cm^–1^ region.

***^13^C solid-state NMR***: AVANCE 500 MHz wide bore spectrometer (BrukerTM) operating at a frequency of 500.12 MHz for 1H and 188.5 MHz for 13C.

Note: The peaks that appeared at 21 and 36-38 ppm in the NMR profiles (Figure 2b, Figure S4, and Figure S6) originated from some trapped N, N-Dimethylacetamide solvent molecules in the porous matrix during washing.

***Thermogravimetric analyses (TGA)***: TGA was carried out on a PerkinElmer Simultaneous Thermalanalyzer STA 6000 under N_2_ atmosphere at a heating rate of 15 ºC min^–1^ within a temperature range of 30-900°C.

***Gas adsorption***: Porosity measurements were conducted on a Micromeritics 3-Flex gas sorption analyzer. Before the measurement, a weighed amount of the sample (~50-100 mg) was degassed at 80 °C for 12 h. Adsorption isotherms were measured in the volumetric method at 273 K (maintained ice-water bath) for CO_2_ adsorption.

***Scanning Electron Microscopy (SEM)***: The JEOL JSM-7610F FEG-SEM and Helios SEM were used for the SEM analysis. It combines an electron column with semi-in-lens detectors and an in-the-lens Schottky field emission gun to deliver ultrahigh-resolution with a wide range of probe currents (1pA to more than 200 nA).

***Transmission Electron Microscopy (TEM)***: The TEM images were recorded by FEI Tecnai TEM 20 kV. The samples were dispersed in acetonitrile solvent for 20-minute sonication and drop cast on copper grids TEM Window (TED PELLA, INC. 200 mesh).

***X-ray photoelectron spectroscopy (XPS):*** 1) XPS studies over the fresh samples were conducted using a ESCALAB Thermo Scientific Theta Probe Angle-Resolved X-ray Photoelectron Spectrometer (East Grinstead, UK). A monochromatic Al Kα X-ray source (hv = 1486.6 eV) was used. The survey spectra were collected using a pass energy of 300 eV, whereas a pass energy of 50 eV was used for acquiring high-resolution core-level spectra. [For samples: Cu-TpAzo, Cu-TpTta, TpAzo, TpTta]

2) X-ray photoelectron spectroscopy (XPS) measurement was carried out by a VG Microtech, model ESCA 3000 instrument equipped with ion gun (EX-05) for cleaning the surface. [For samples: Cu-Tp, Tp, TpaAzo+Cu]

***Solid-state UV-visible spectra***: The solid-state UV-visible spectral analysis was carried out using The LAMBDA 1050 UV/Vis/NIR spectrometer.


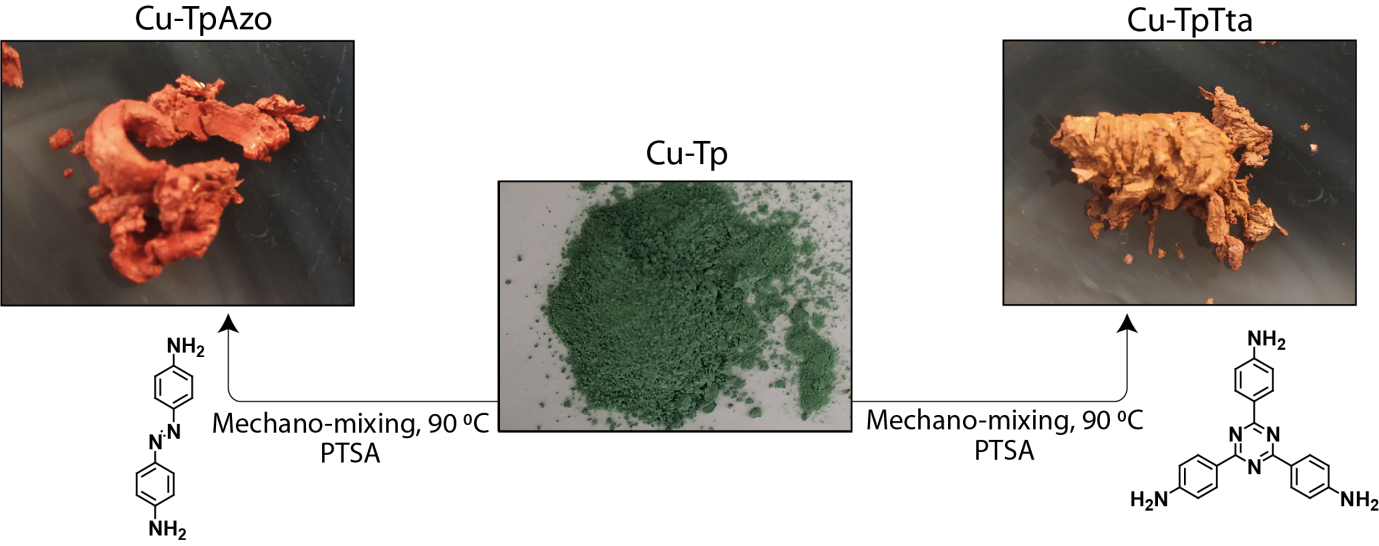


Figure S1: The digital photographs of Cu-Tp, Cu-TpAzo and Cu-TpTta.


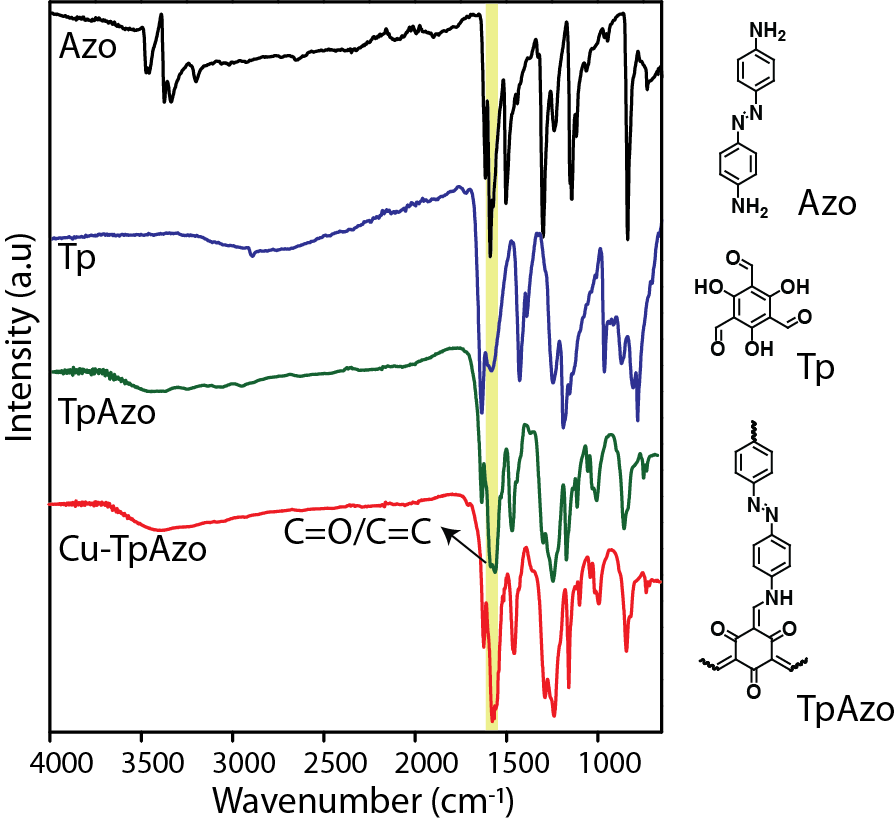


Figure S2: The FT-IR spectra of Cu-TpAzo, TpAzo, and monomers.


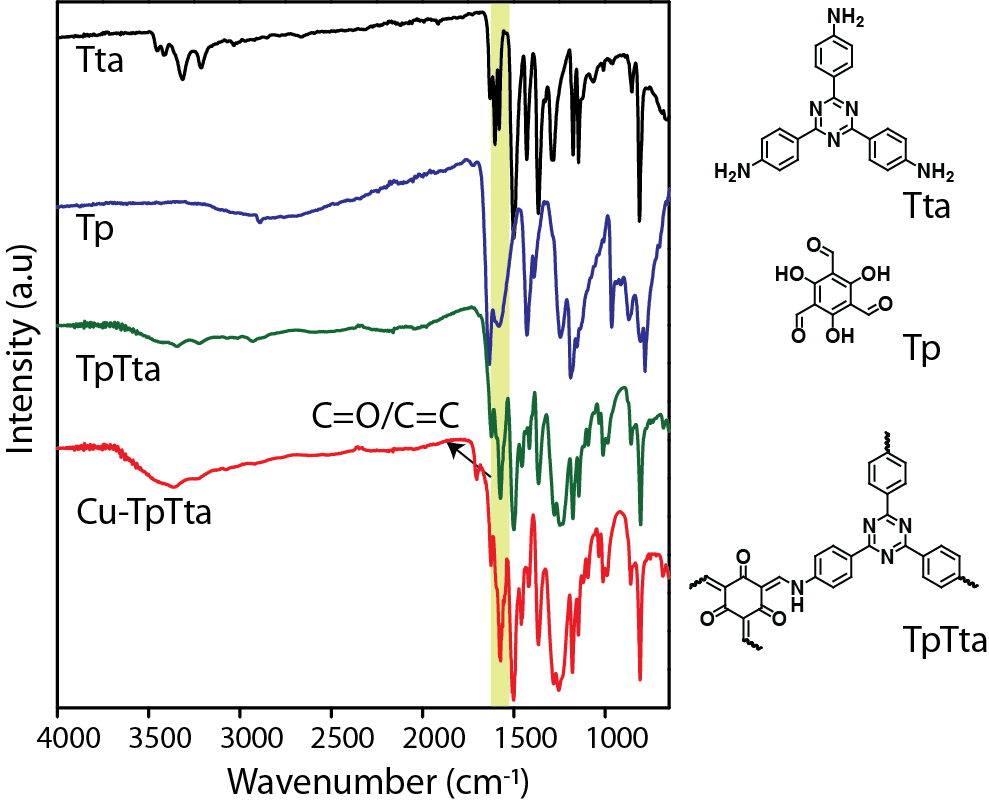


Figure S3: The FT-IR spectra of Cu-TpTta, TpTta and monomers.


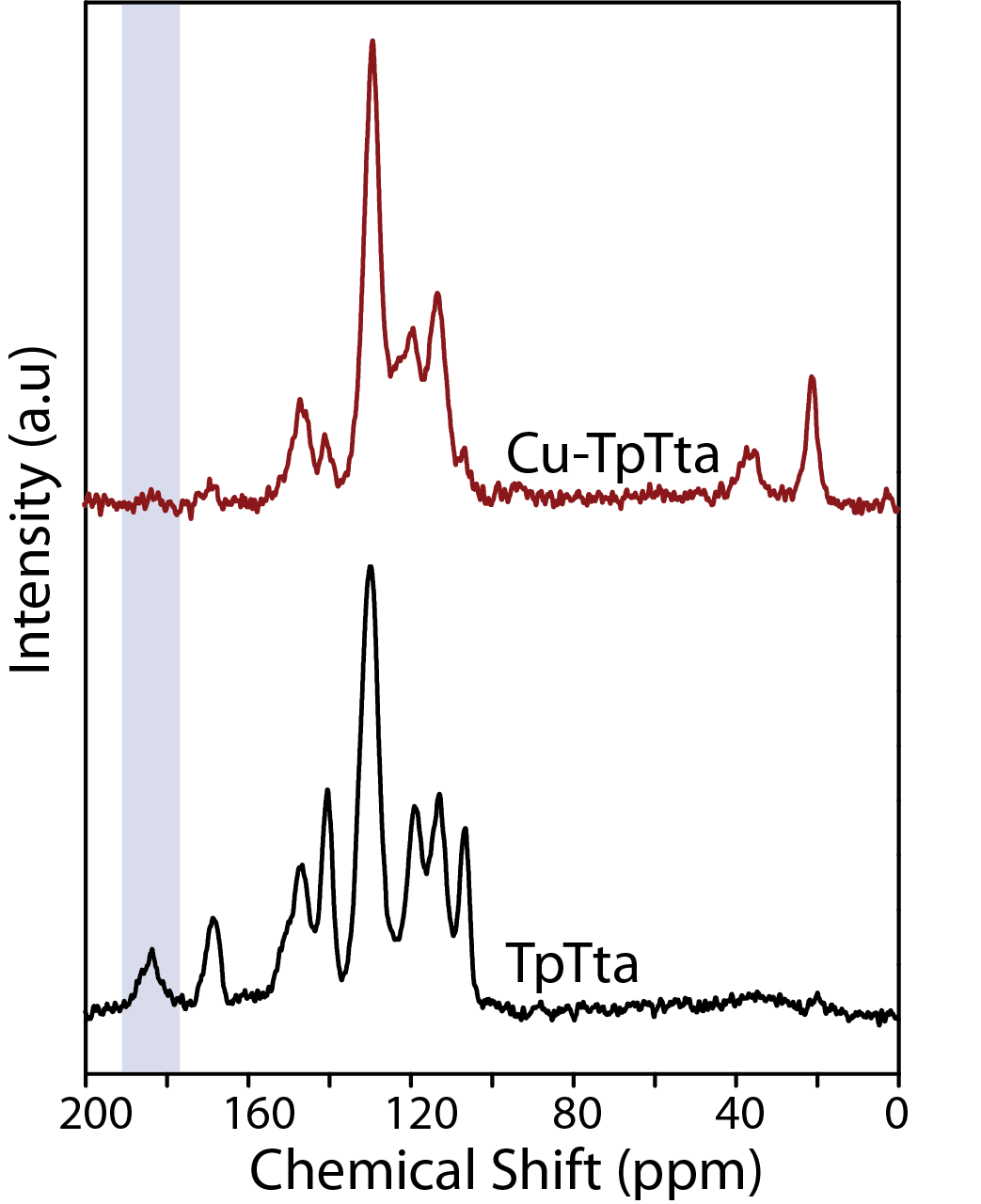


Figure S4: The ^13^C NMR spectra of Cu-TpTta and TpTta.


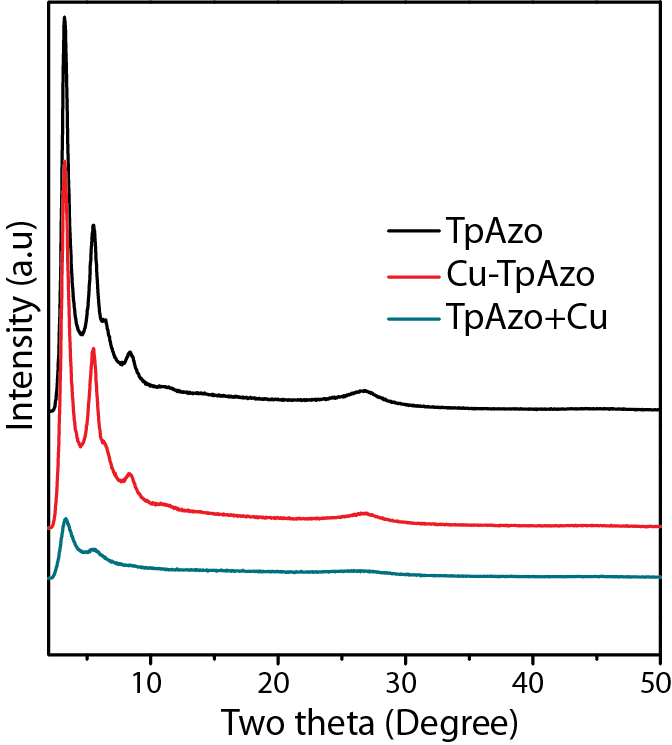


Figure S5: The PXRD profiles of TpAzo, Cu-TpAzo and TpAzo+Cu.


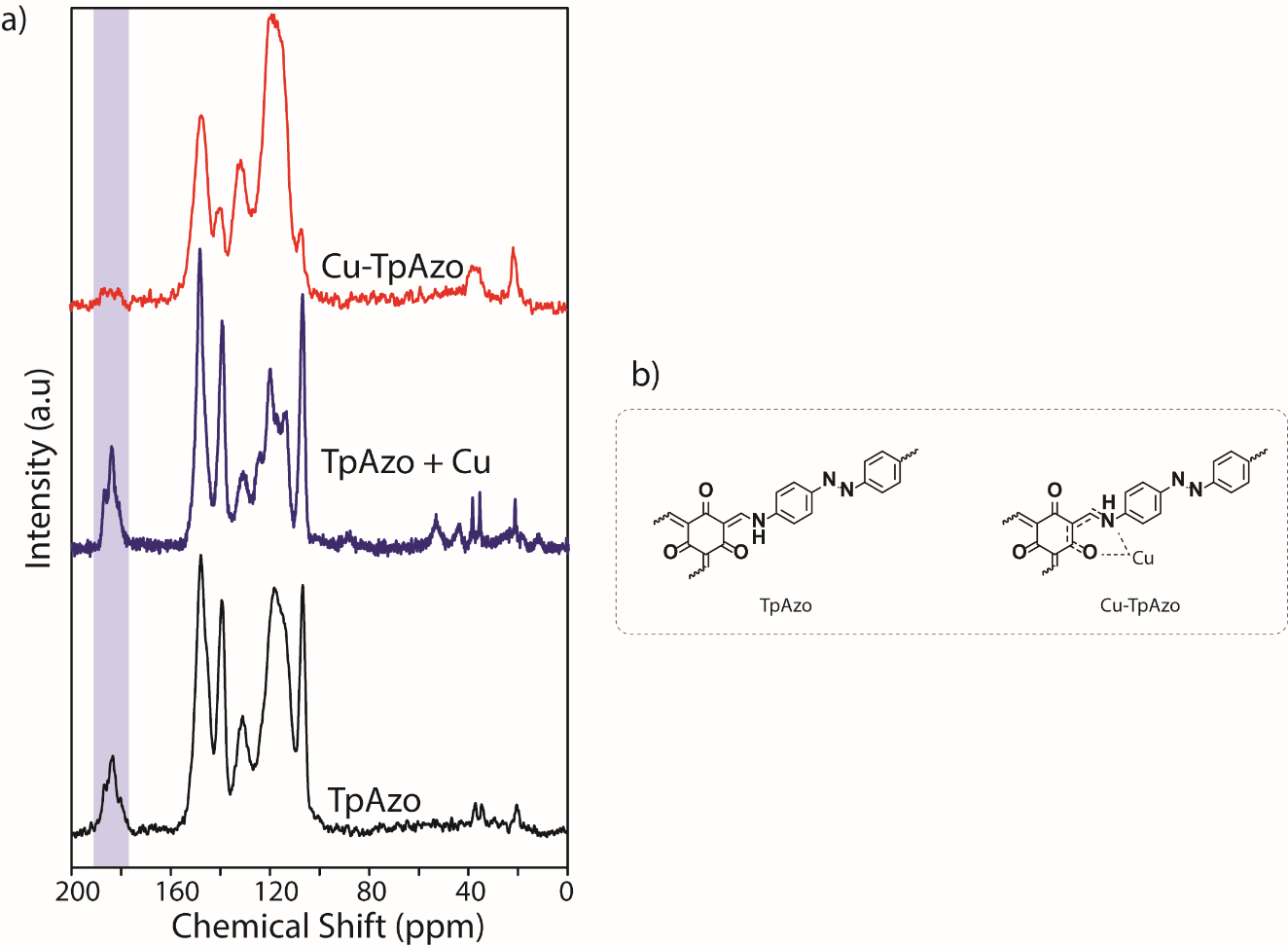


Figure S6: a) The ^13^C NMR spectra of Cu-TpAzo, TpAzo, TpAzo+Cu. b) The possible copper interaction with TpAzo.

Note:


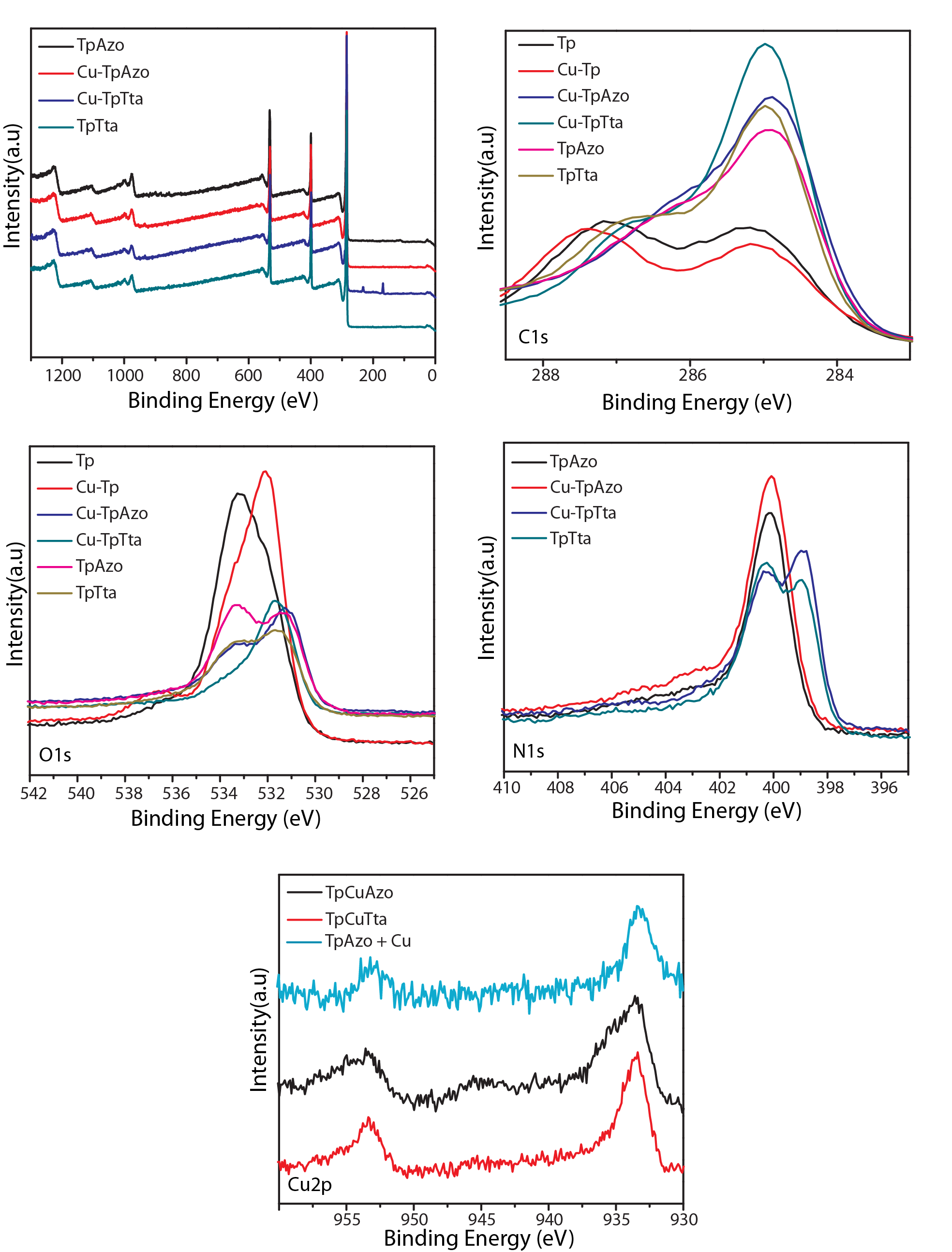


Figure S7: The XPS profiles of all materials.


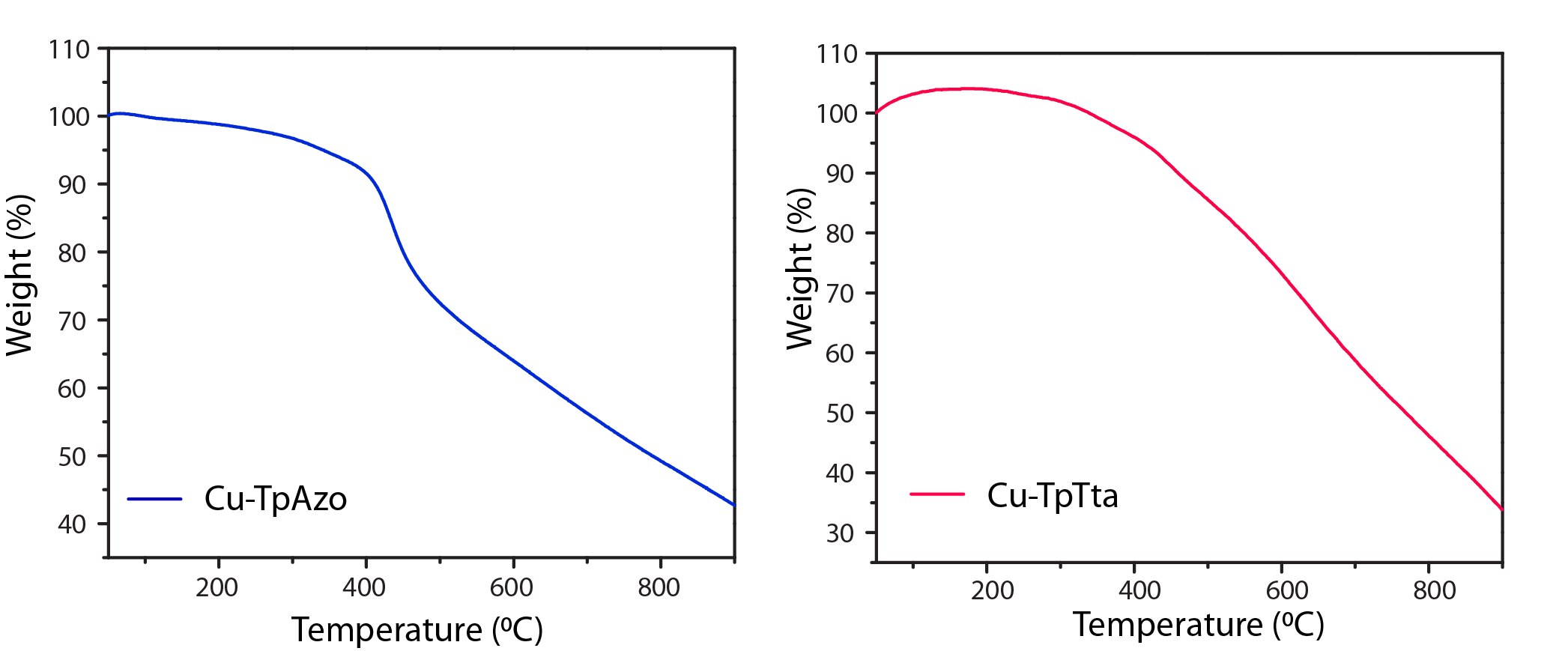


**Figure S8**: The TGA profiles of CuTp-Azo and Cu-TpTta.


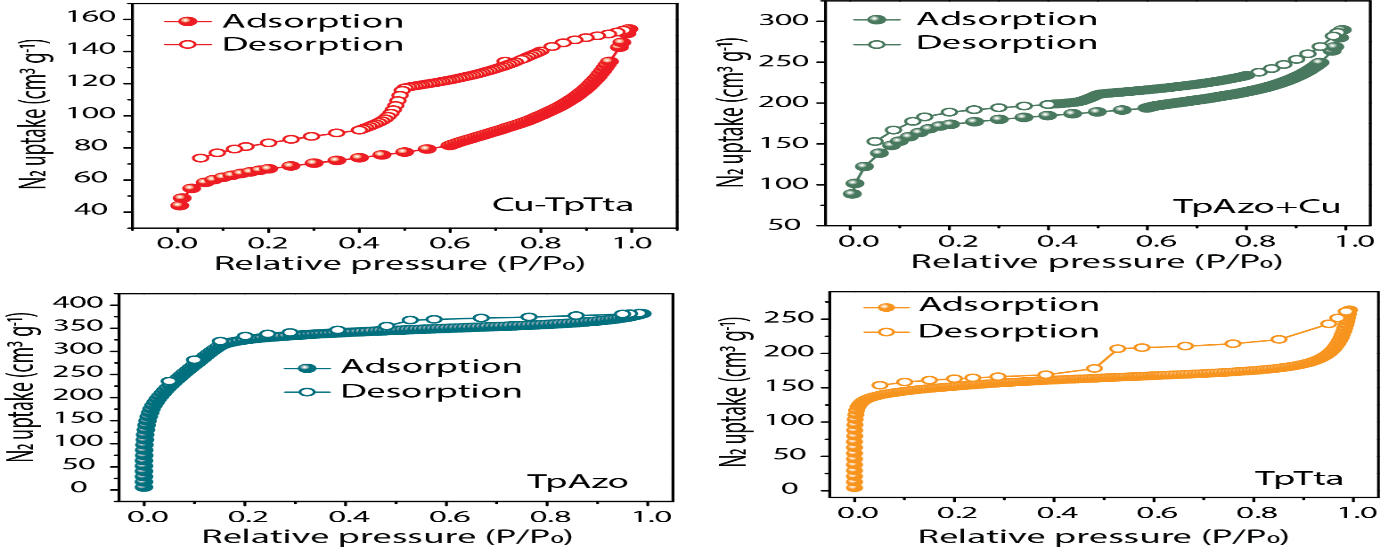


**Figure S9**: The N_2_ gas adsorption isotherm profiles of Cu-TpTta and TpAzo+Cu.


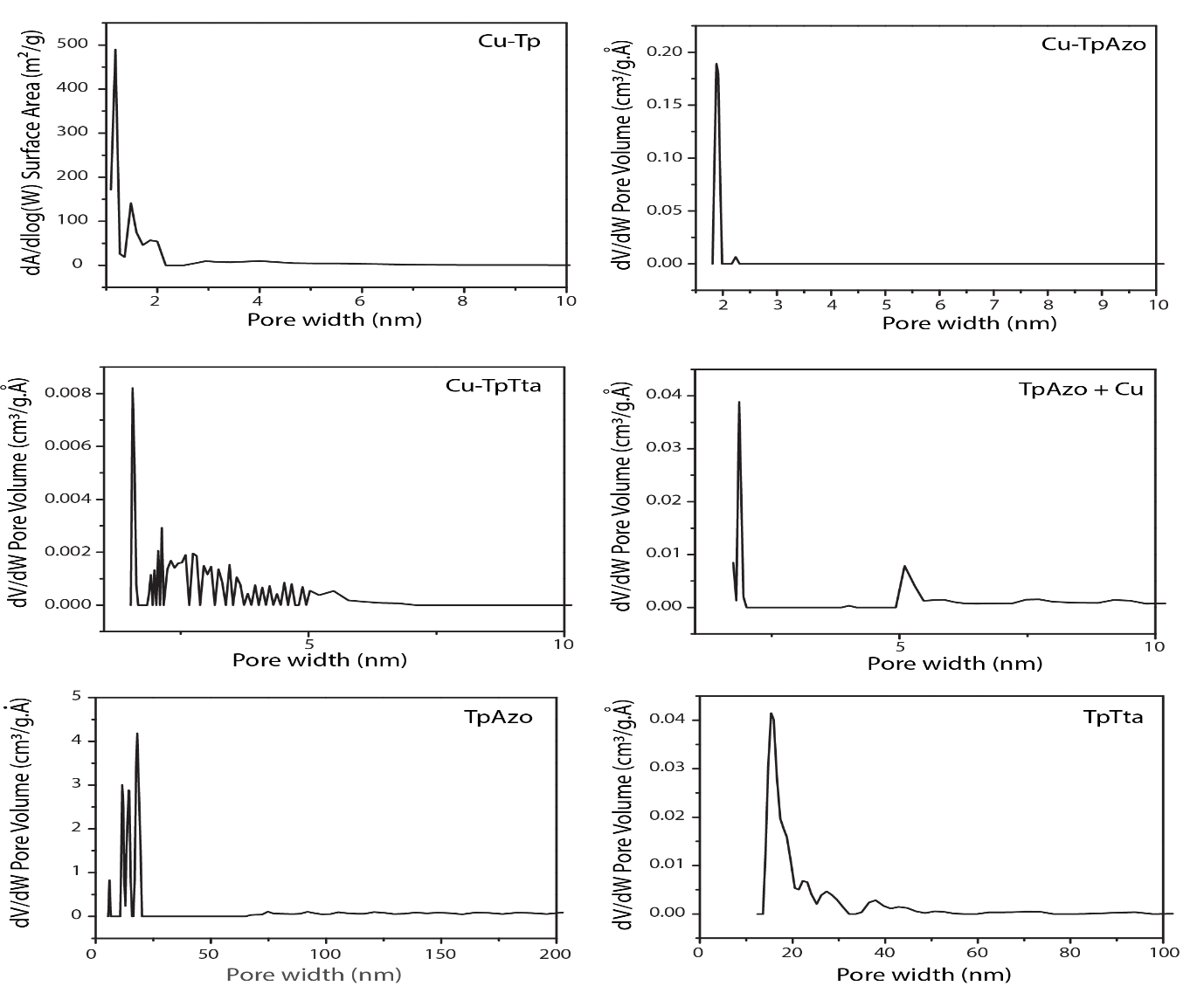


**Figure S10**: The NLDFT pore size distributions of Cu-Tp, Cu-TpAzo, Cu-TpTta and TpAzo+Cu.

| **COF** | **Surface area (m^2^g^-1^)** | **Pore volume (Single point adsorption) cm³ g^-1^** | **Pore size**  **nm** |
| --- | --- | --- | --- |
| Cu-TpAzo | 1065 | 1.04 | 1.9 |
| TpAzo | 971 | 0.59 | 1.9 |
| TpAzo+Cu | 266 | 0.34 | 1.9 |
| Cu-TpTta | 214 | 0.23 | 1.56 |
| TpTta | 578 | 0.41 | 1.56 |

**Table S1**: The porosity features comparison table of COFs.


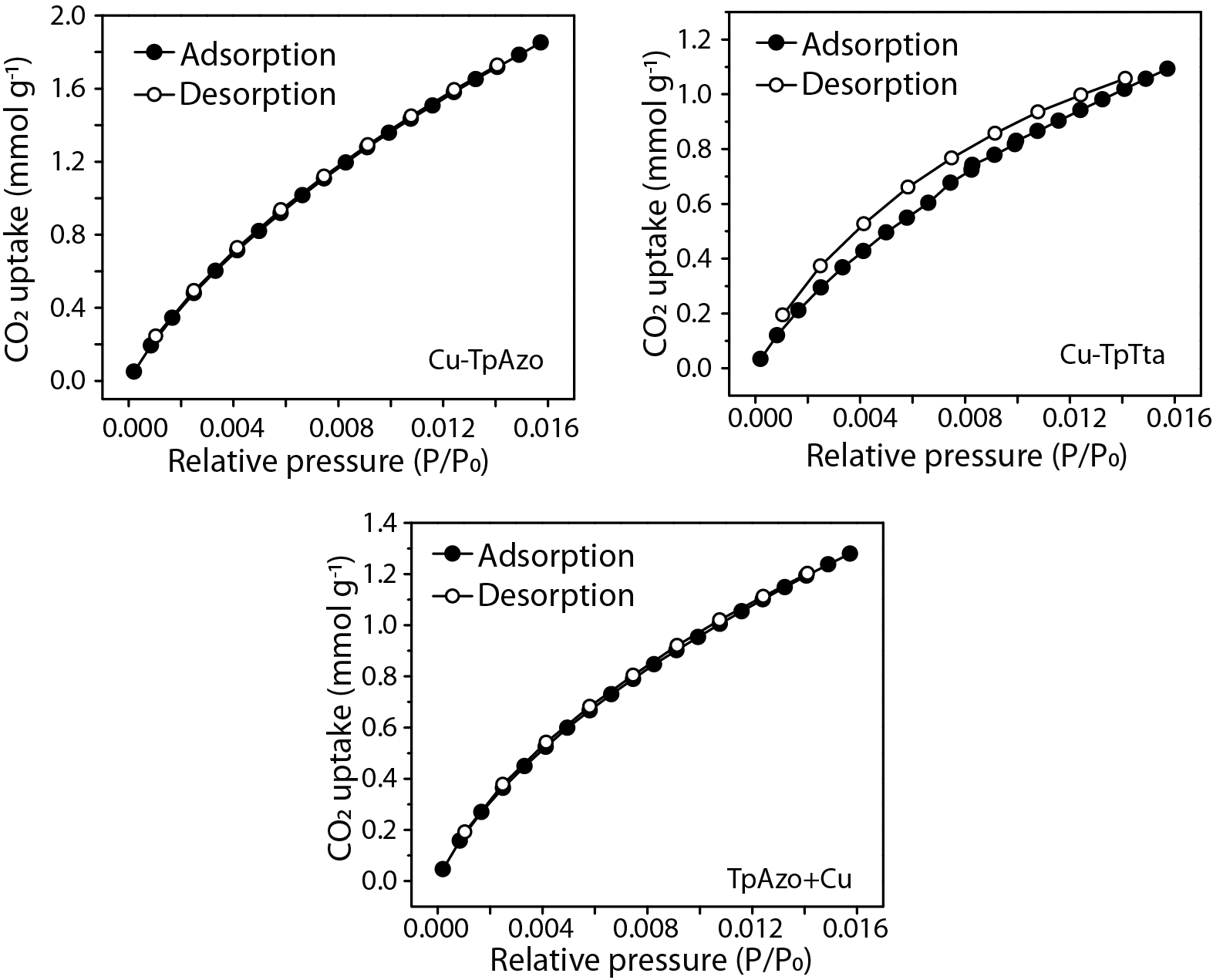


**Figure S11**: The CO_2_ sorption isotherm profiles Cu-TpAzo, Cu-TpTta and TpAzo+Cu.


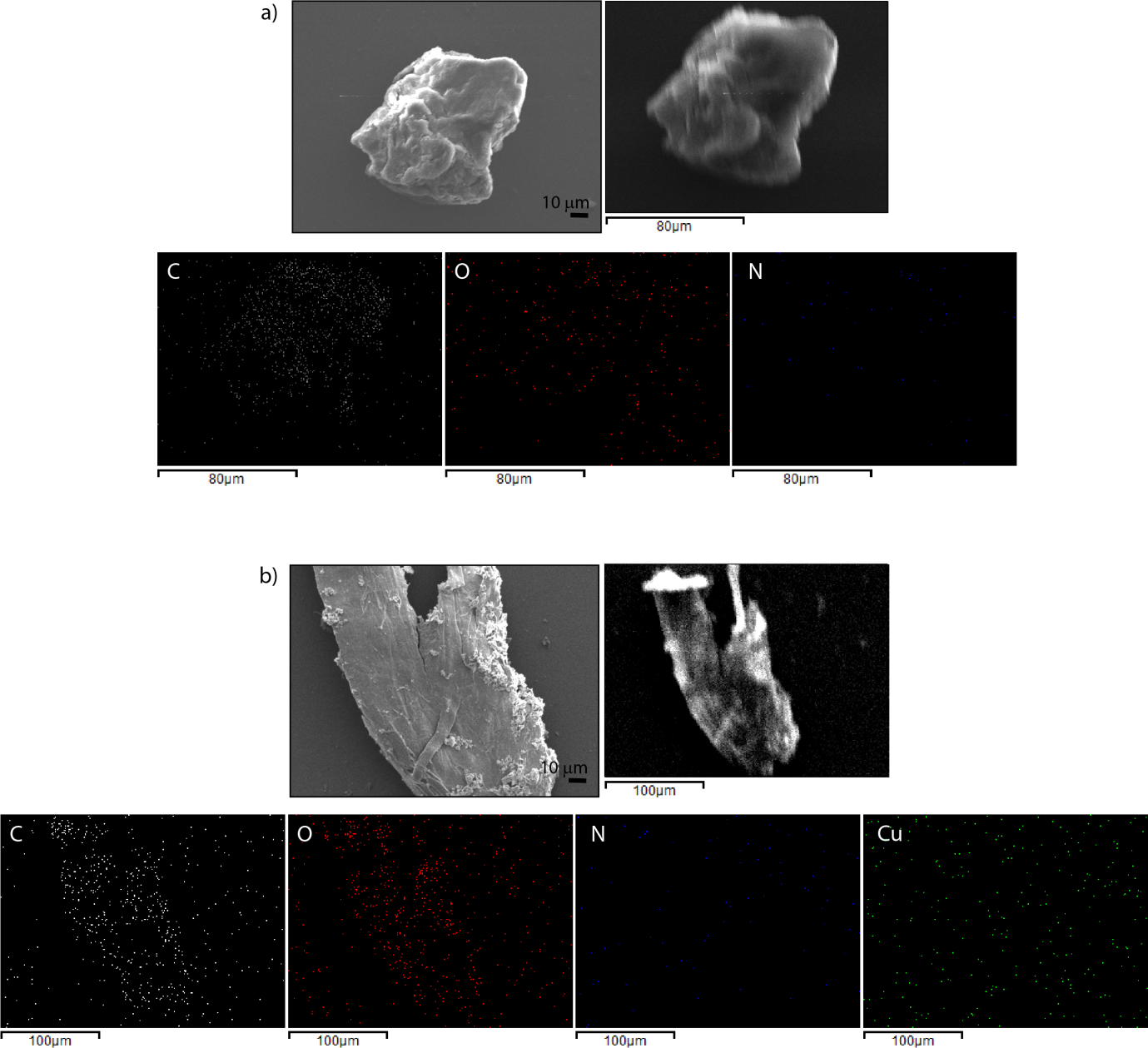


Figure S12: SEM images and elemental mapping of a) TpAzo and b) Cu-TpAzo.


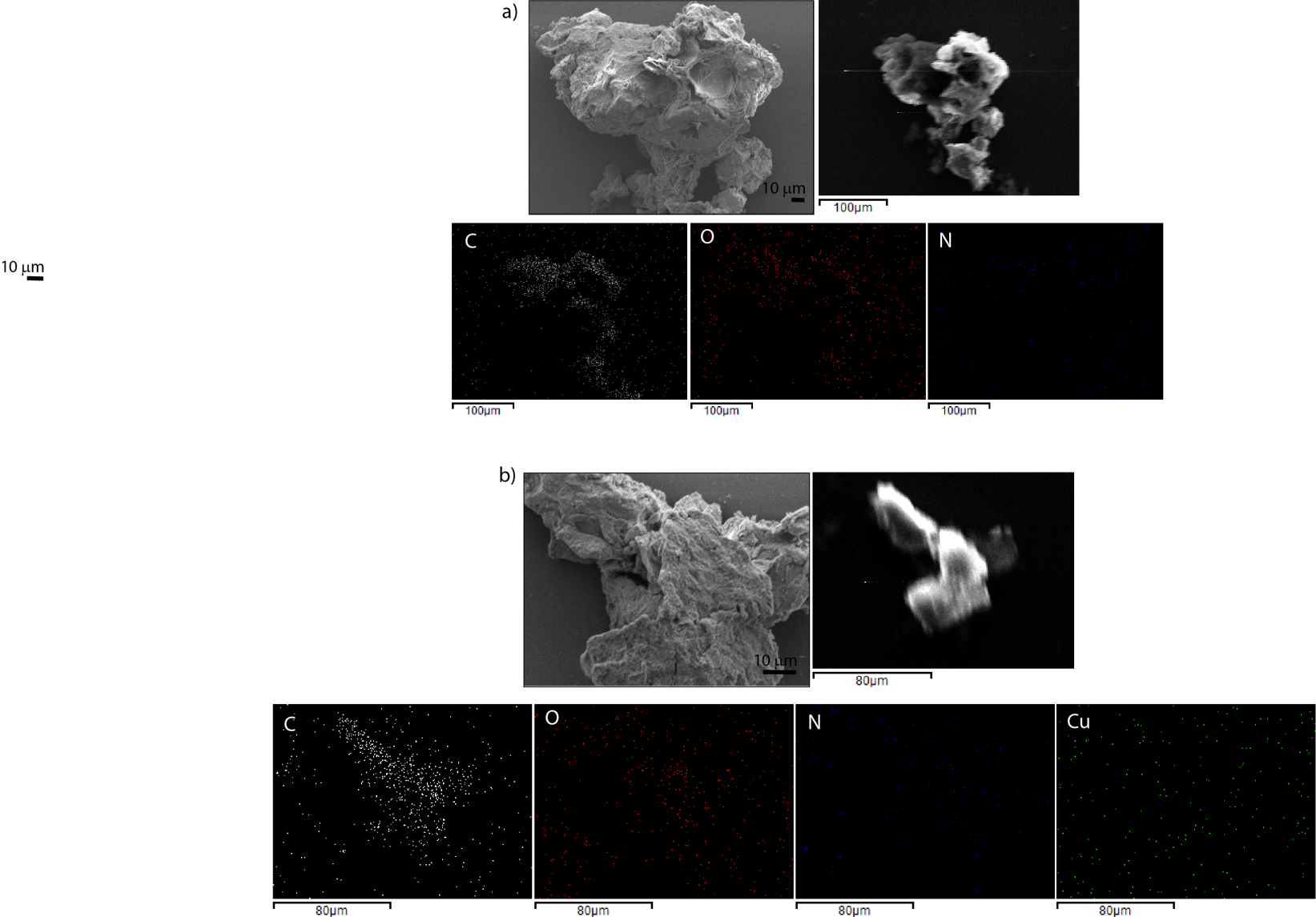


Figure S13: SEM images and elemental mapping of a) TpTta and b) Cu-TpTta.


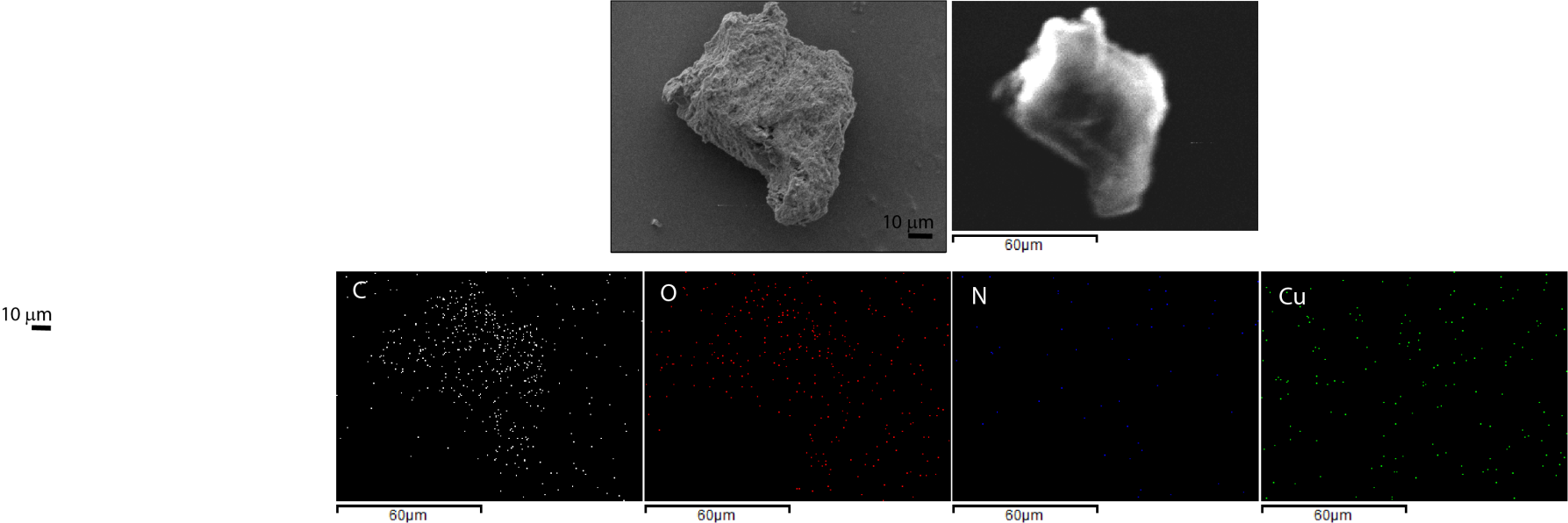


Figure S14: SEM images and elemental mapping of TpAzo+Cu.


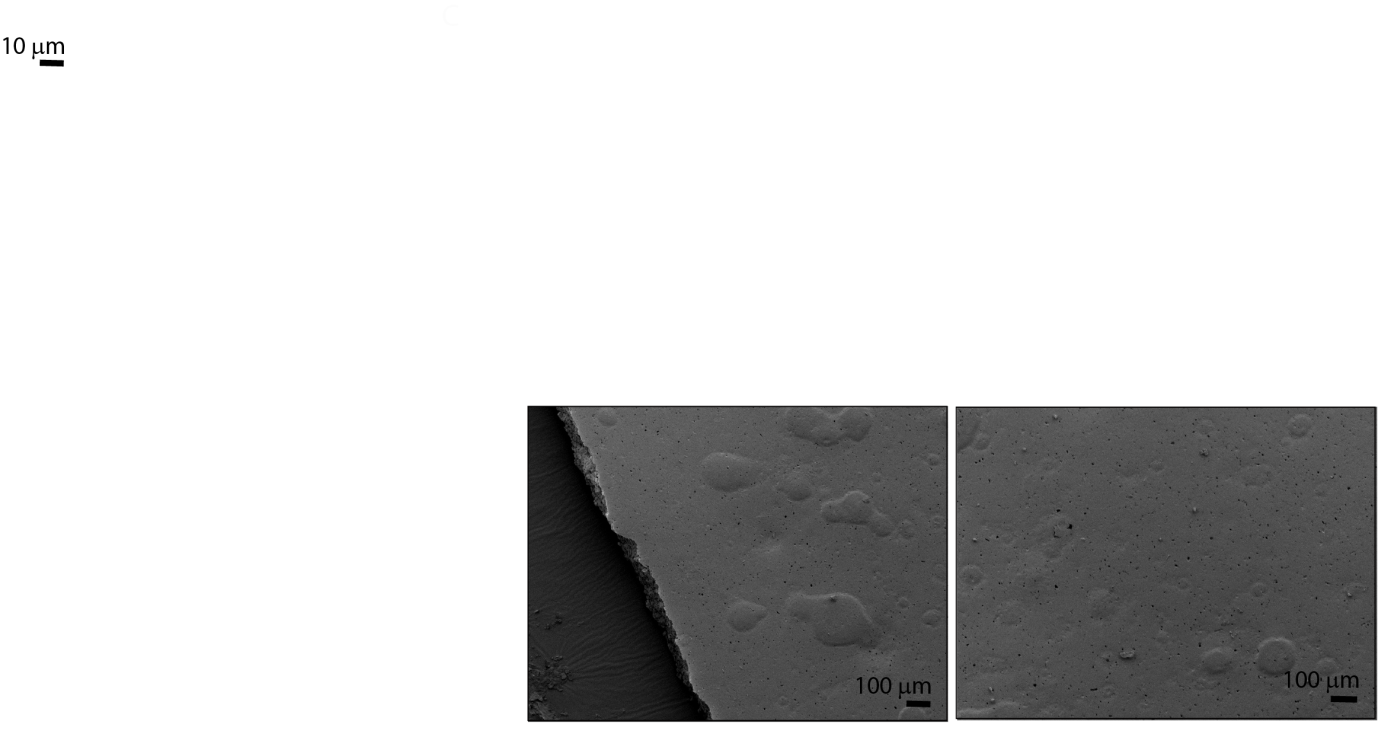


Figure S15: SEM images and elemental mapping of Cu-TpAzo free-standing sheet.


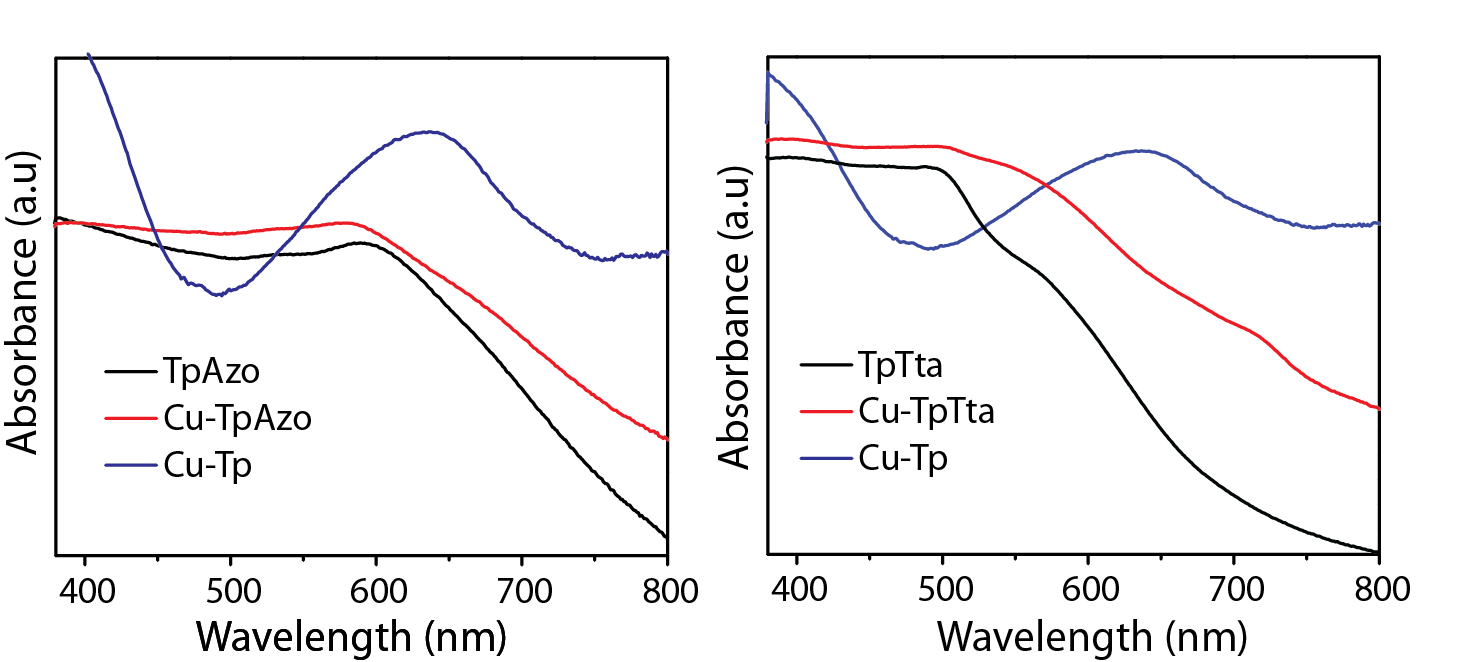


Figure S16: Solid-state UV-Visible spectra of Cu-Tp, TpAzo, TpTta, Cu-TpAzo and Cu-TpTta.


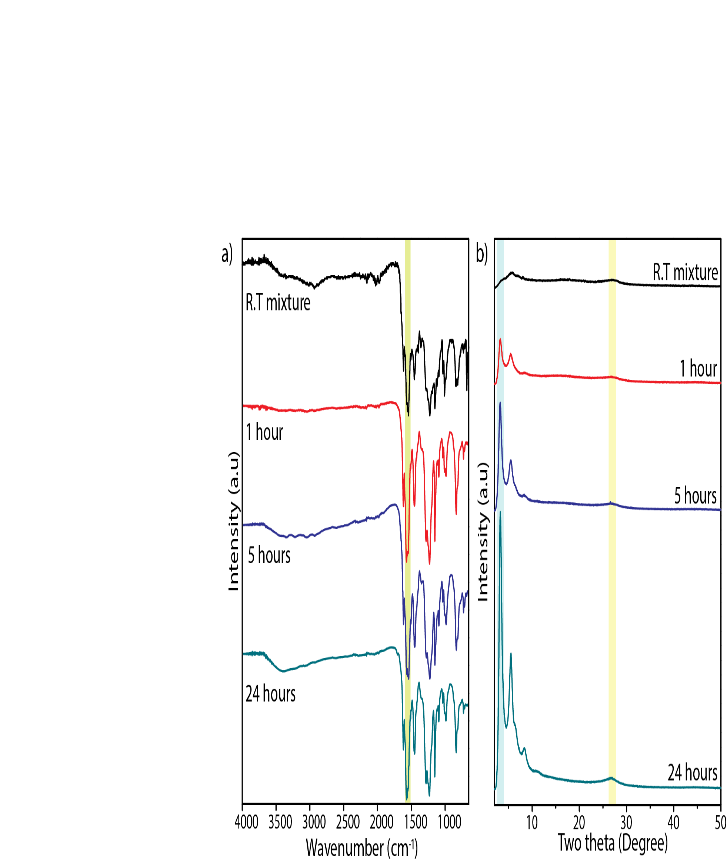


Figure S17: a) FT-IR spectra and b) PXRD profiles of time-controlled study of Cu-TpAzo.


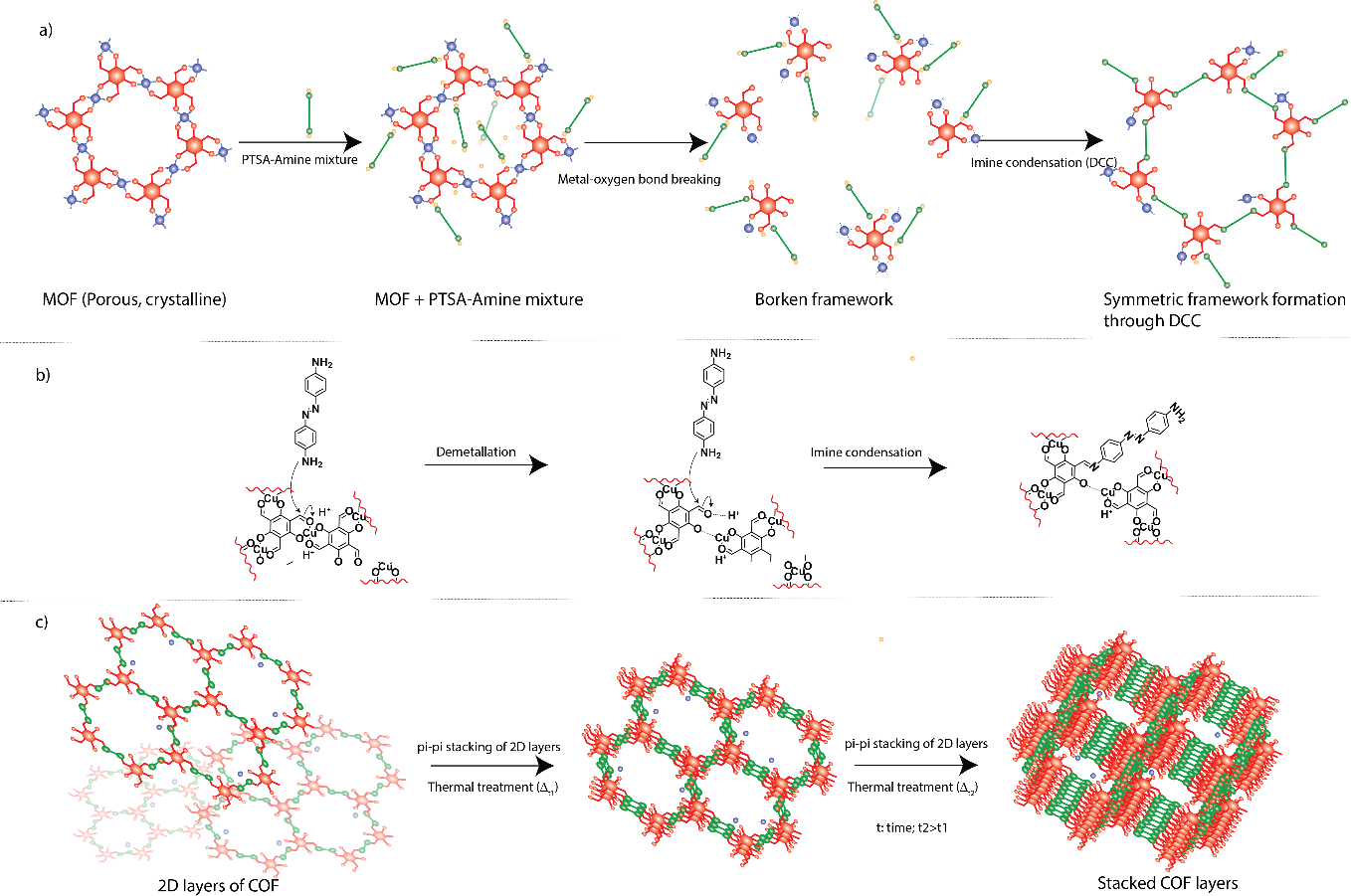


Figure S18: The possible mechanism of the formation of COF from MOF.

| **Condition** | **Intensity (counts) of 100 peak (at 2ϴ 3.2°)** | **Intensity (counts) of 100 peak (at 2ϴ 26.7°)** | **Ratio of the peak intensities (100/001)** |
| --- | --- | --- | --- |
| Room temperature | NIL | 1542 | ------ |
| 1 hour at 90 °C | 7816 | 1343 | 5.81 |
| 5 hours at 90 °C | 18734 | 1338 | 14.00 |
| 24 hours at 90 °C | 51354 | 2880 | 17.8 |

Table S2: The relative peak intensity ratio of 1 0 0/ 0 0 1 upon thermal treatment time.

**Possible mechanism of MOF to COF formation:**

*1) Chemical bond formation:*

Step 1: In Cu-Tp MOF, both aldehyde and phenolic oxygen atoms in Tp are chemically bonded to copper. The PTSA-Amine mixture breaks this bond especially at aldehyde oxygen-copper through nucleophilic amine interaction at electrophilic carbon (Figure S18 a & b).

Step 2: The above electrophile-nucleophile interaction with the help of protons leads to imine condensation reaction.

Step 3: The imine condensation reaction disrupts the Cu-Tp structure completely and converts it to 2D layers of COFs through dynamic covalent chemistry (DCC). The DCC allowed the organized and symmetric formation of 2D frameworks.

*2) Structure formation:*

Step 1: The FT-IR profile of room temperature mixture of Cu-Tp and PTSA-Amine showed the formation of the polymerization of COF (Figure S17a). Furthermore, PXRD profile indicates the formation of 2D layers (Figure S17b). Although new COF chemical bonds are formed at room temperature, the PXRD profile clearly shows the absence of the first peak (1 0 0) which originated from the electron density across the stacking.

Step 2: The 2D layers of COF are stacked through pi-pi interaction and form periodic nanochannels. The ex-situ PXRD analysis showed the gradual increment of crystallinity of COF upon increasing the time of thermal treatment at 90 °C. The relative peak intensity of 100 has been improved from 1 hour to 24 hours of thermal treatment signifies the role of temperature for the effective stacking of the formed 2D COF layers (Table S1).


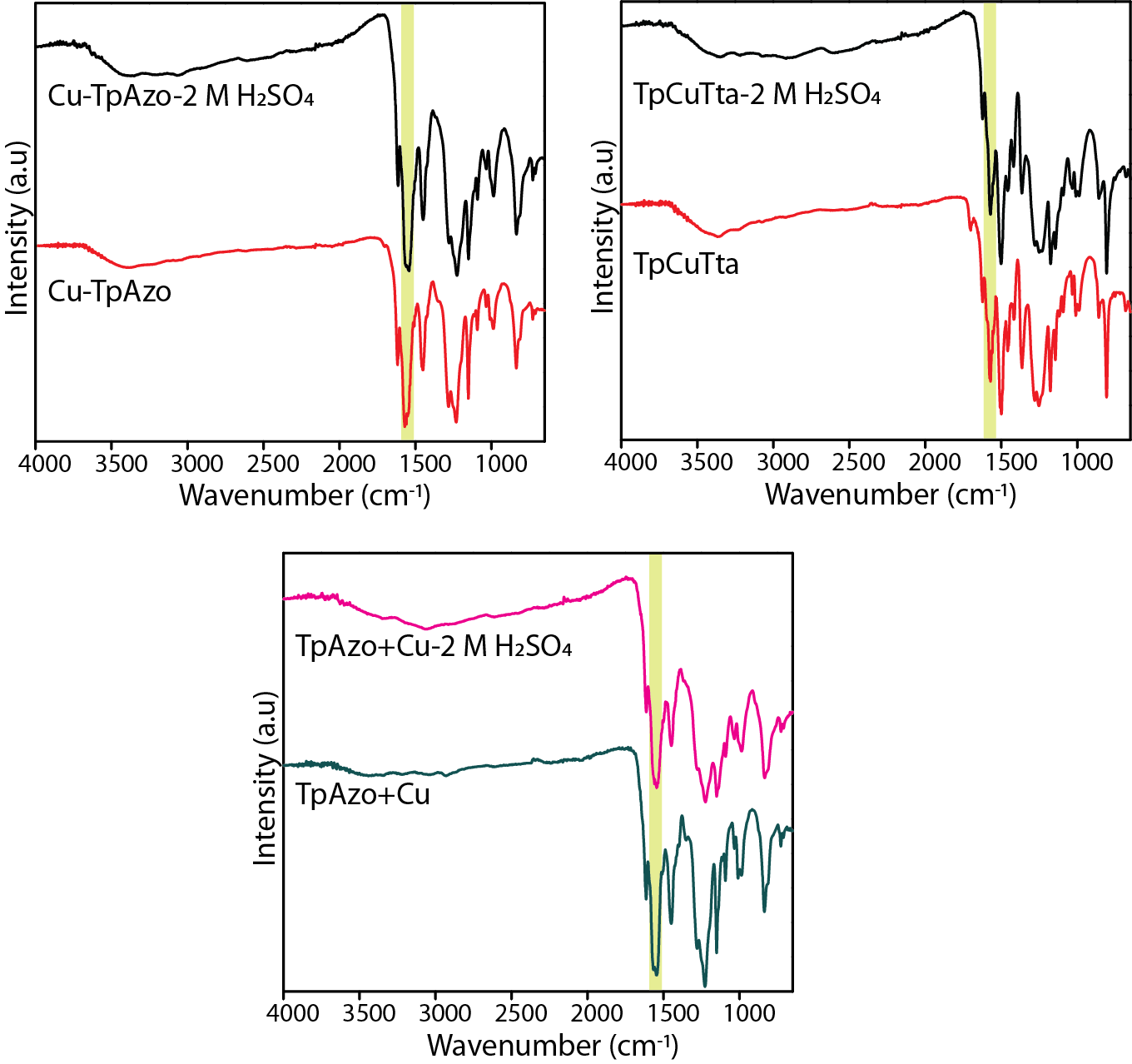


Figure S19: FT-IR profiles of Cu-TpAzo, Cu-TpTta and TpAzo+Cu after 2M H_2_SO_4_ treatment for 24 hours.


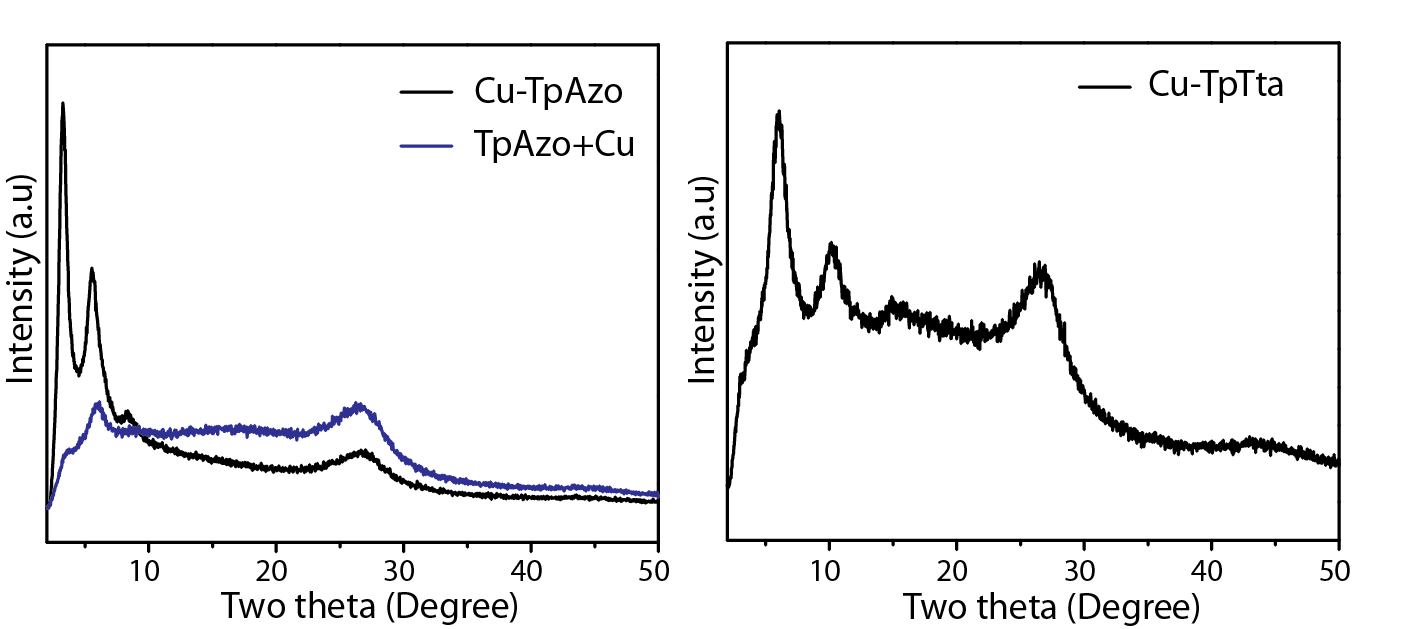


Figure S20: PXRD profiles of Cu-TpAzo, Cu-TpTta, and TpAzo+Cu after 2M H_2_SO_4_ treatment for 24 hours.

.
